# Supplementary figures and images for: PASK links cellular energy metabolism with a mitotic self-renewal network to establish differentiation competence
Source: eLife. 2023 Apr 13;12:e81717. doi: 10.7554/eLife.81717 (PMC10162801; doi:10.7554/eLife.81717)

**Figure 2 – figure supplement 1**

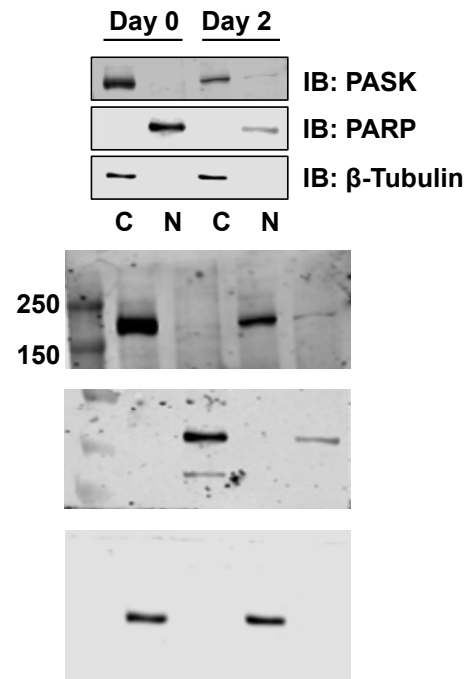

Supplement: Figure 2—figure supplement 1—source data 1. [file elife-81717-fig2-figsupp1-data1.zip › Figure 2 - Source Data 1/Figure 2 - Figure supplement 1 - Source Data.pdf]

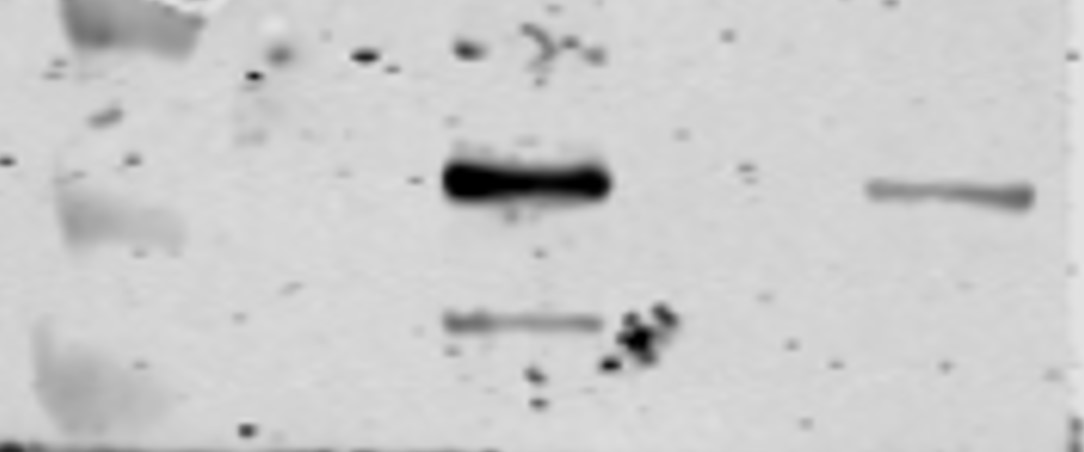

Supplement: Figure 2—figure supplement 1—source data 1. [file elife-81717-fig2-figsupp1-data1.zip › Figure 2 - Source Data 1/parp.jpg]

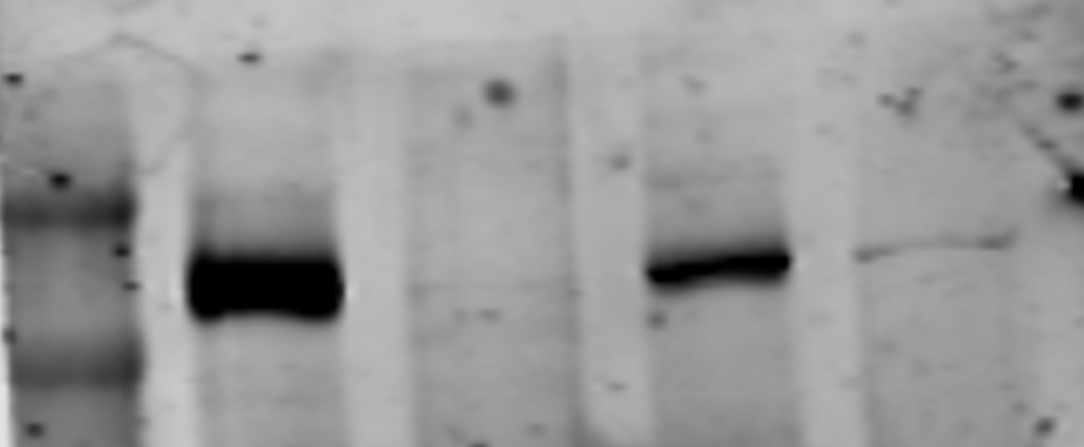

Supplement: Figure 2—figure supplement 1—source data 1. [file elife-81717-fig2-figsupp1-data1.zip › Figure 2 - Source Data 1/pask.jpg]

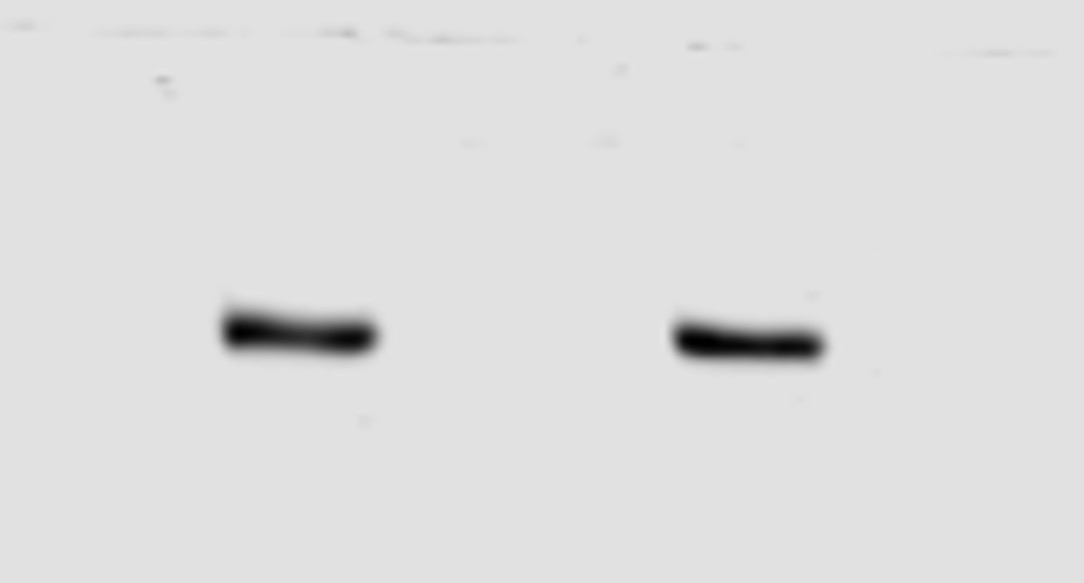

Supplement: Figure 2—figure supplement 1—source data 1. [file elife-81717-fig2-figsupp1-data1.zip › Figure 2 - Source Data 1/tub.jpg]

**Figure 3 – Source Data Table 1**


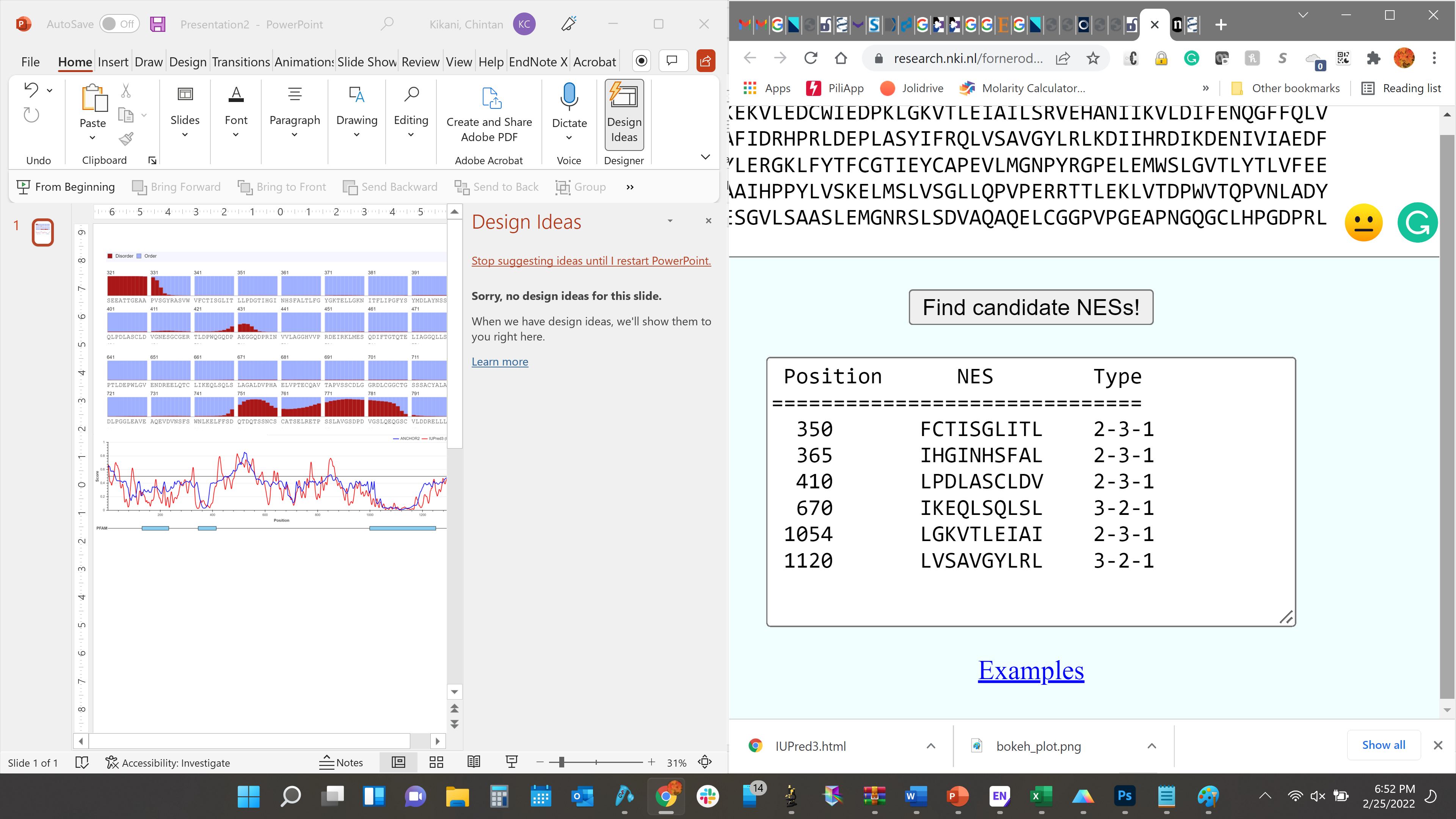

Supplement: Figure 3—source data 1. [file elife-81717-fig3-data1.zip › Figure 3 - Source Data 1/Figure 3 - Figure supplement 1.docx]

**Figure 3 – Source Data Table 2**

**
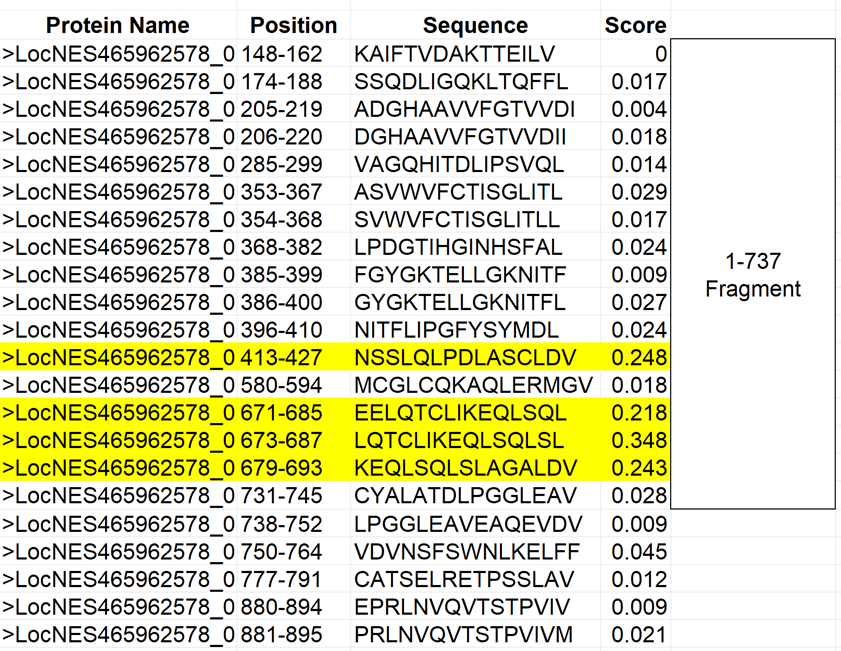
**

Supplement: Figure 3—source data 2. [file elife-81717-fig3-data2.zip › Figure 3 - Source Data 2/Figure 3 - Figure supplement 2.docx]

**Figure 4B**

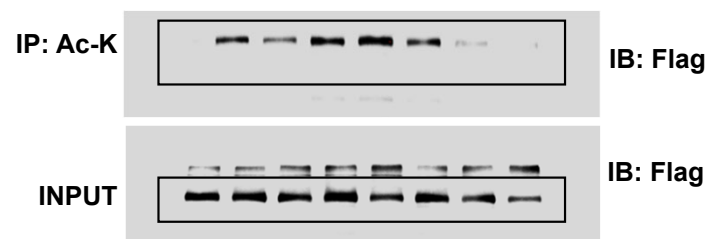

Supplement: Figure 4—source data 1. [file elife-81717-fig4-data1.zip › Figure 4 - Source Data 1-4B/Figure 4B - Source Data.pdf]

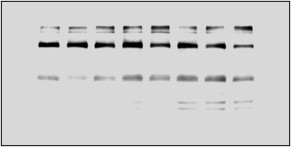

Supplement: Figure 4—source data 1. [file elife-81717-fig4-data1.zip › Figure 4 - Source Data 1-4B/INPUT FLAG.jpg]

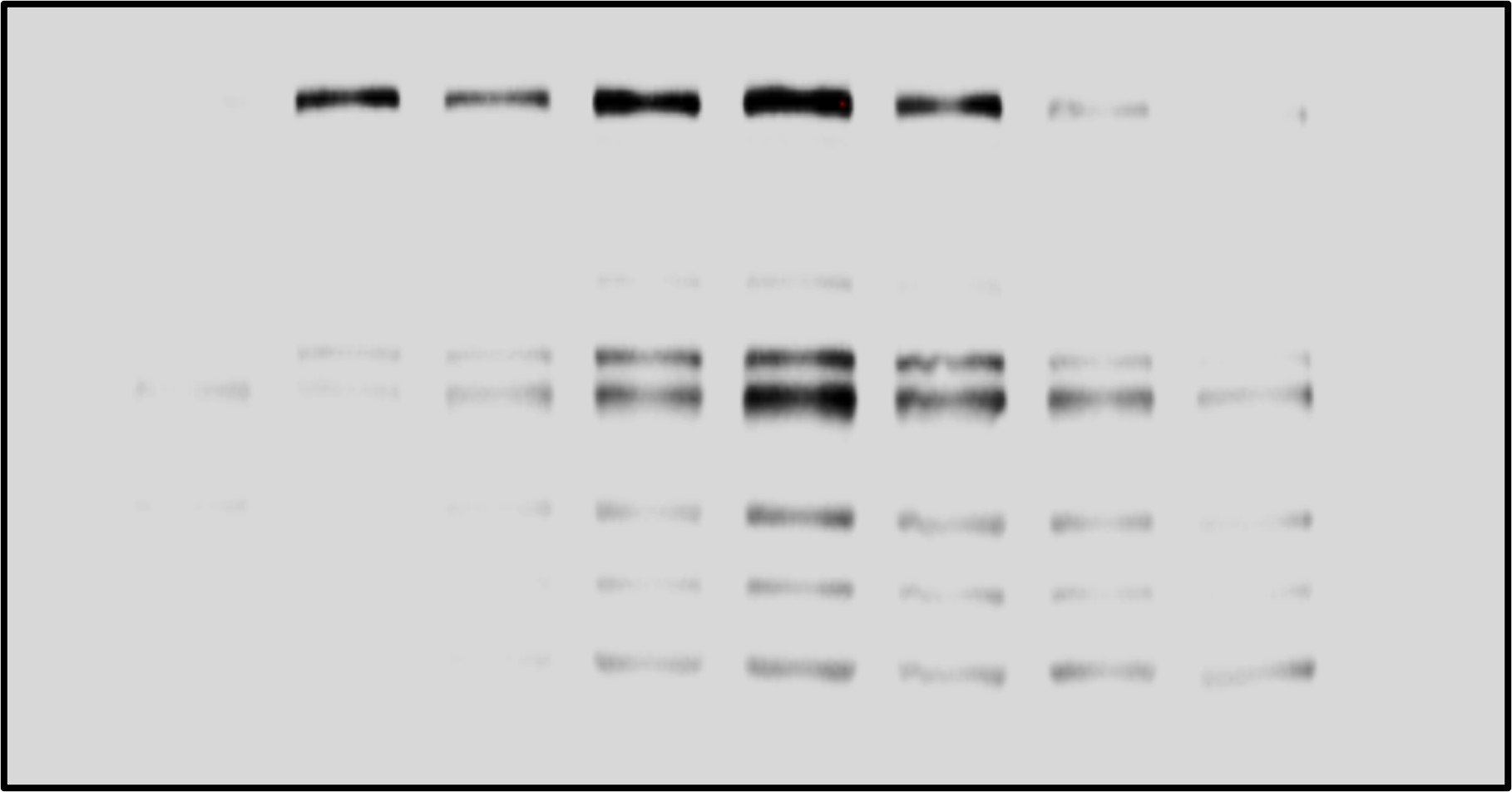

Supplement: Figure 4—source data 1. [file elife-81717-fig4-data1.zip › Figure 4 - Source Data 1-4B/IP AcK anti FLAG.jpg]

**Figure 4C**

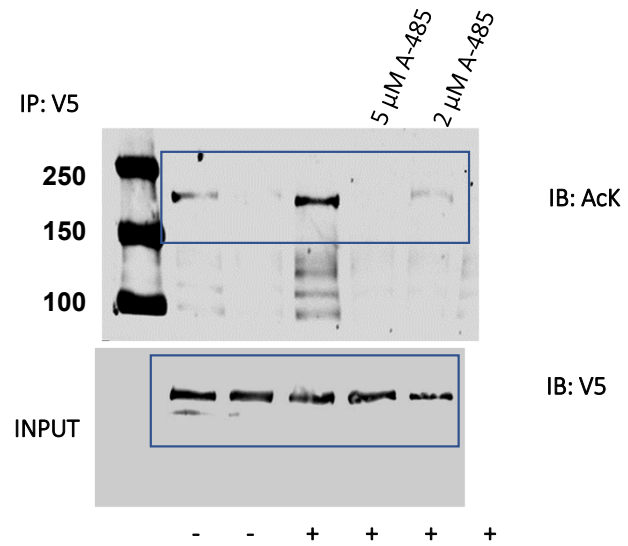

Supplement: Figure 4—source data 2. [file elife-81717-fig4-data2.zip › Figure 4 - Source Data 2 - 4C/Figure 4C - Source Data.pdf]

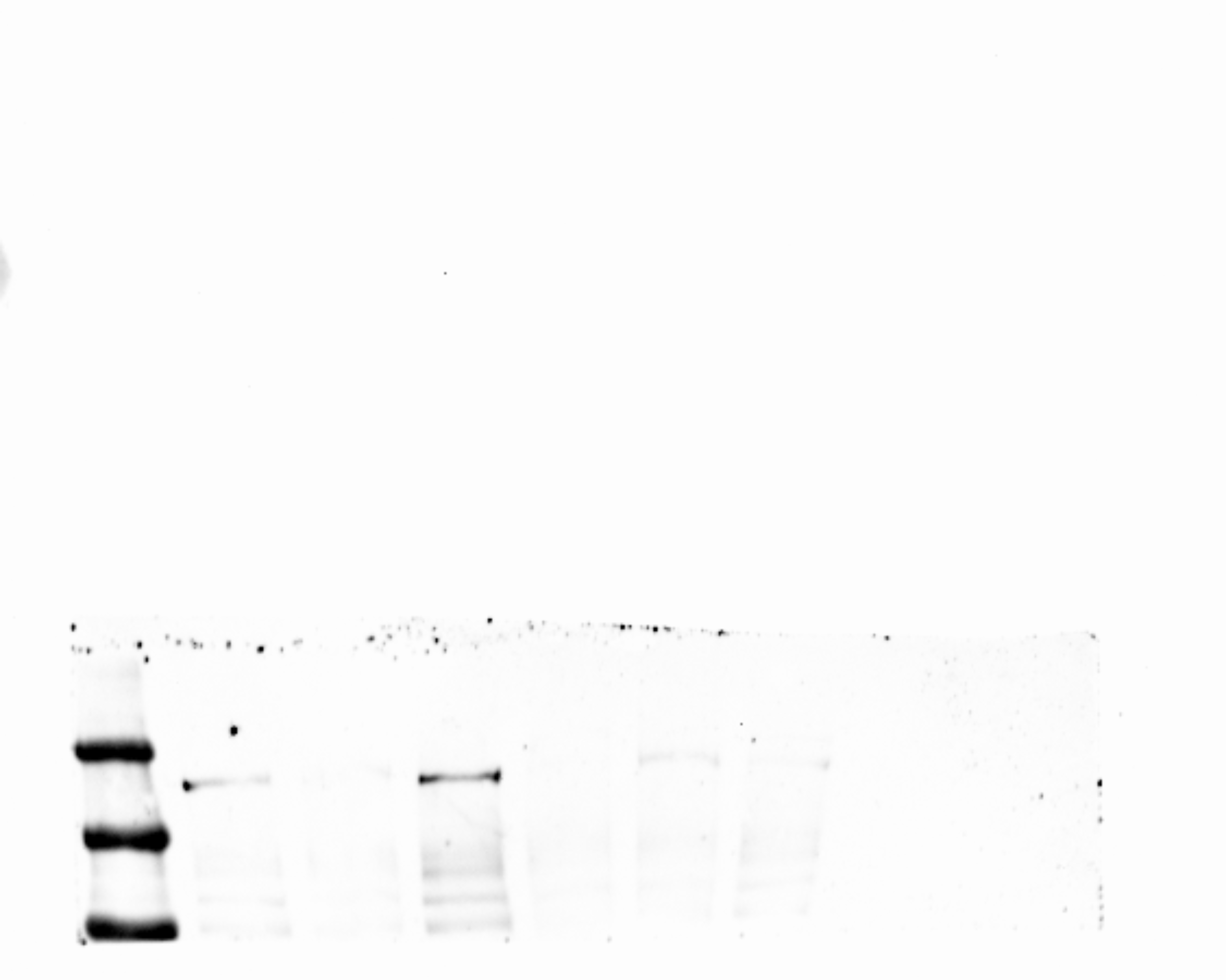

Supplement: Figure 4—source data 2. [file elife-81717-fig4-data2.zip › Figure 4 - Source Data 2 - 4C/IP_V5WB_AcKChemi.tif]

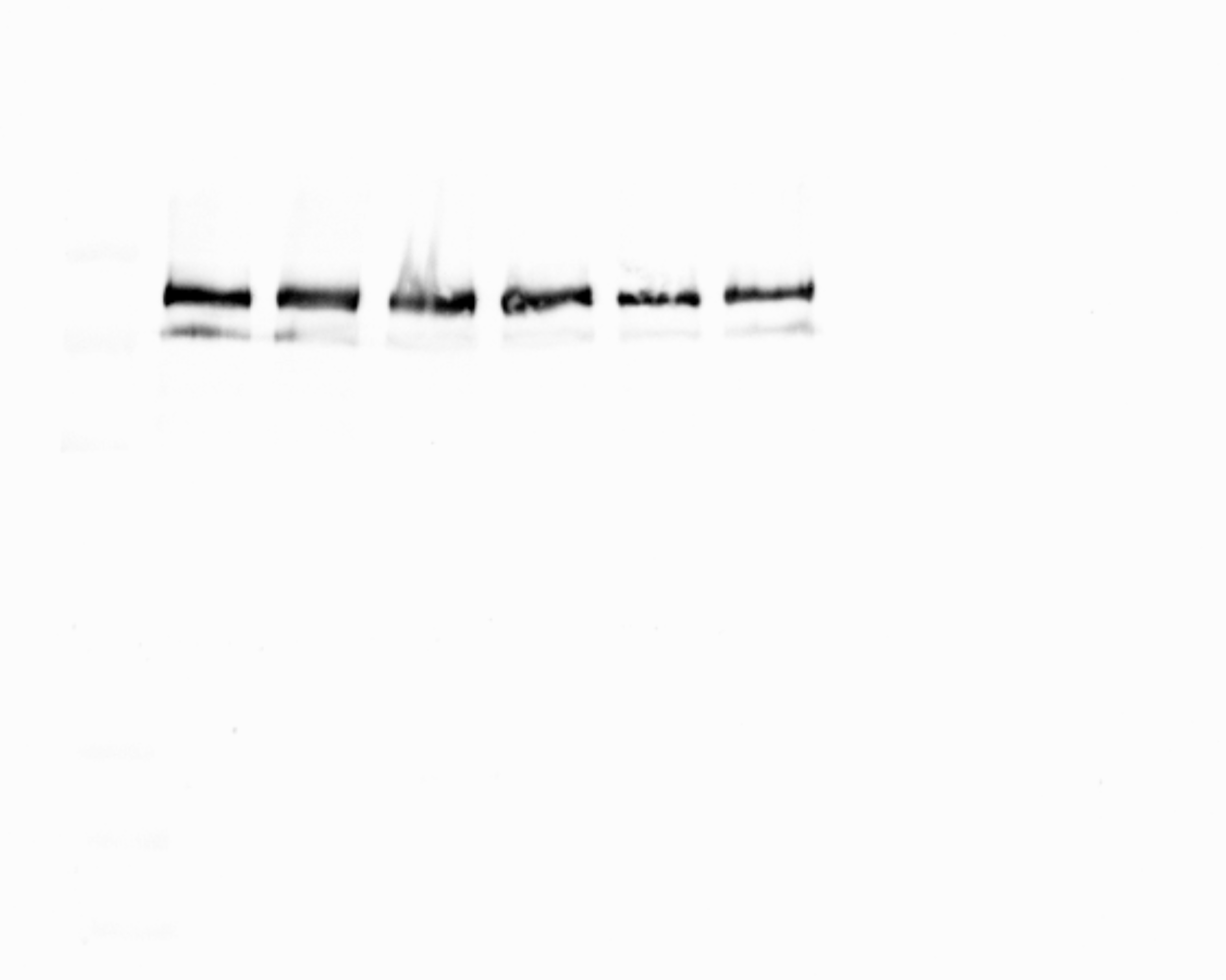

Supplement: Figure 4—source data 2. [file elife-81717-fig4-data2.zip › Figure 4 - Source Data 2 - 4C/V5inputChemi.tif]

**Figure 4F**

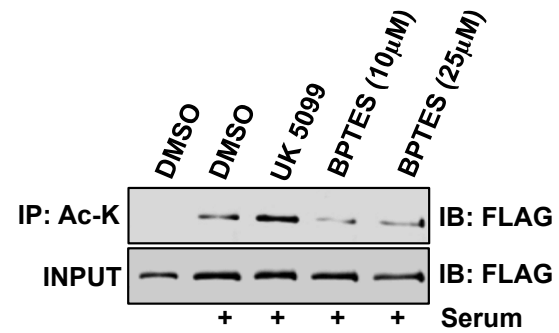

**Source Data**

**Figure 4 – figure  
supplement 1G**

**Figure 4F**

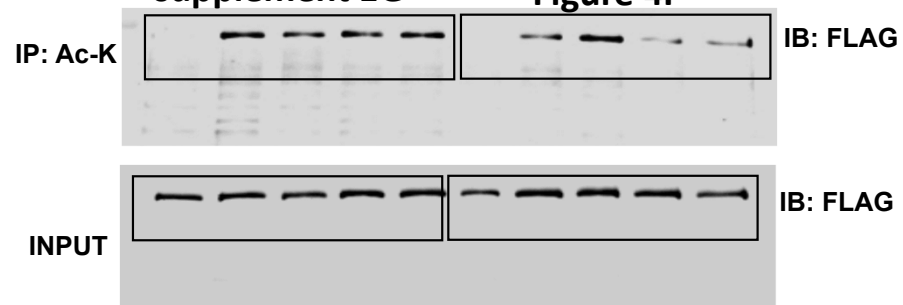

Supplement: Figure 4—source data 3. [file elife-81717-fig4-data3.zip › Figure 4 - Source Data 3 - 4F/Figure 4F - Source Data.pdf]

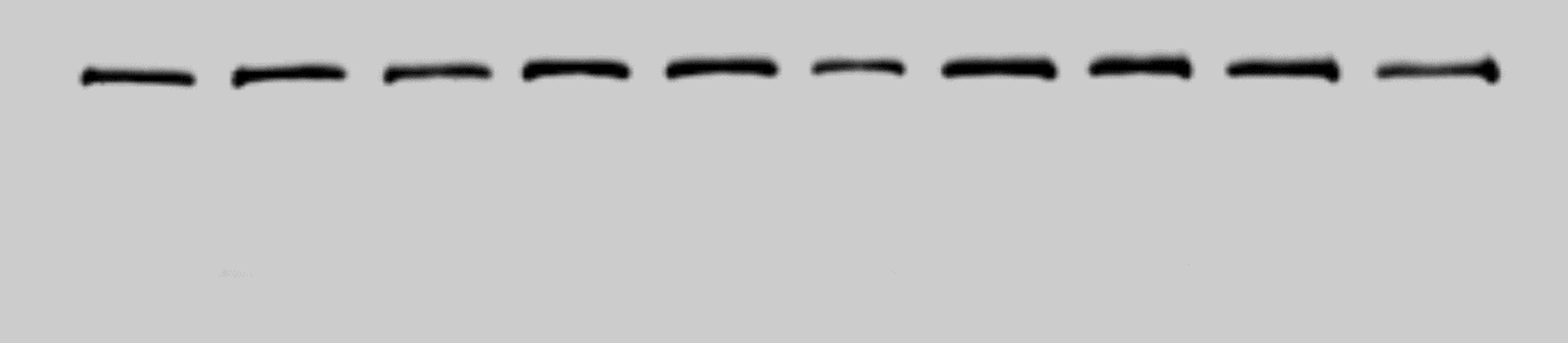

Supplement: Figure 4—source data 3. [file elife-81717-fig4-data3.zip › Figure 4 - Source Data 3 - 4F/Input Flg.jpg]

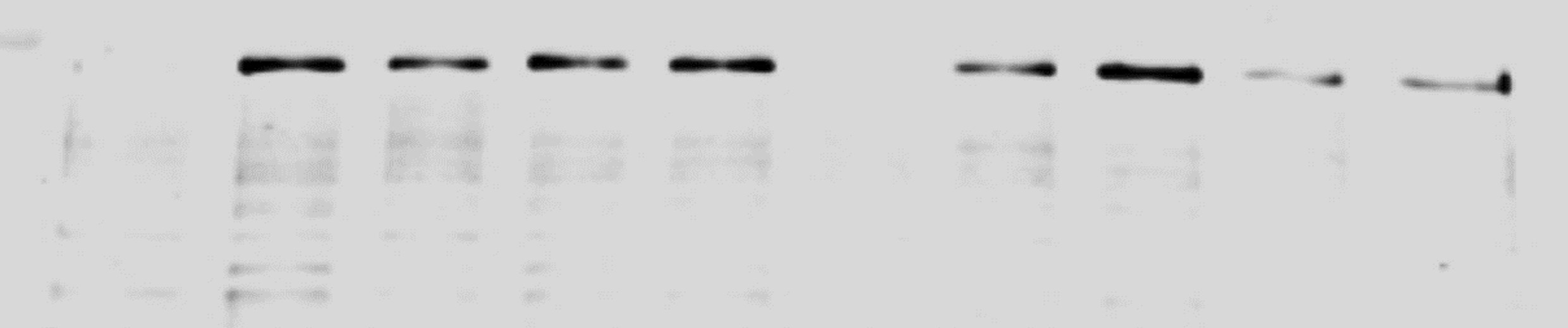

Supplement: Figure 4—source data 3. [file elife-81717-fig4-data3.zip › Figure 4 - Source Data 3 - 4F/IPAckWBFlg.jpg]

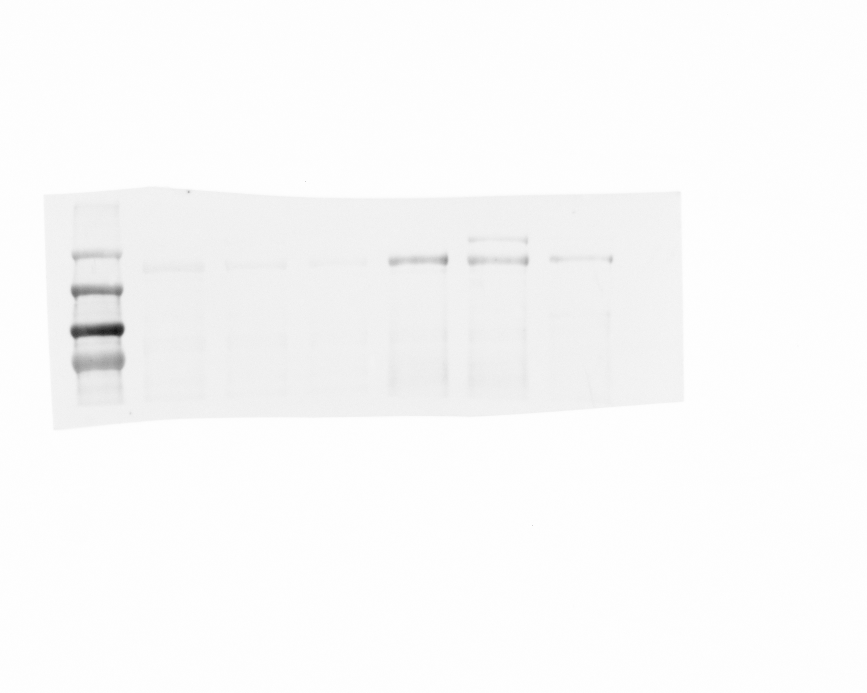

Supplement: Figure 4—source data 4. [file elife-81717-fig4-data4.zip › Figure 4 - Source Data 4 - 4G/Figure 4G - PASK.tif]

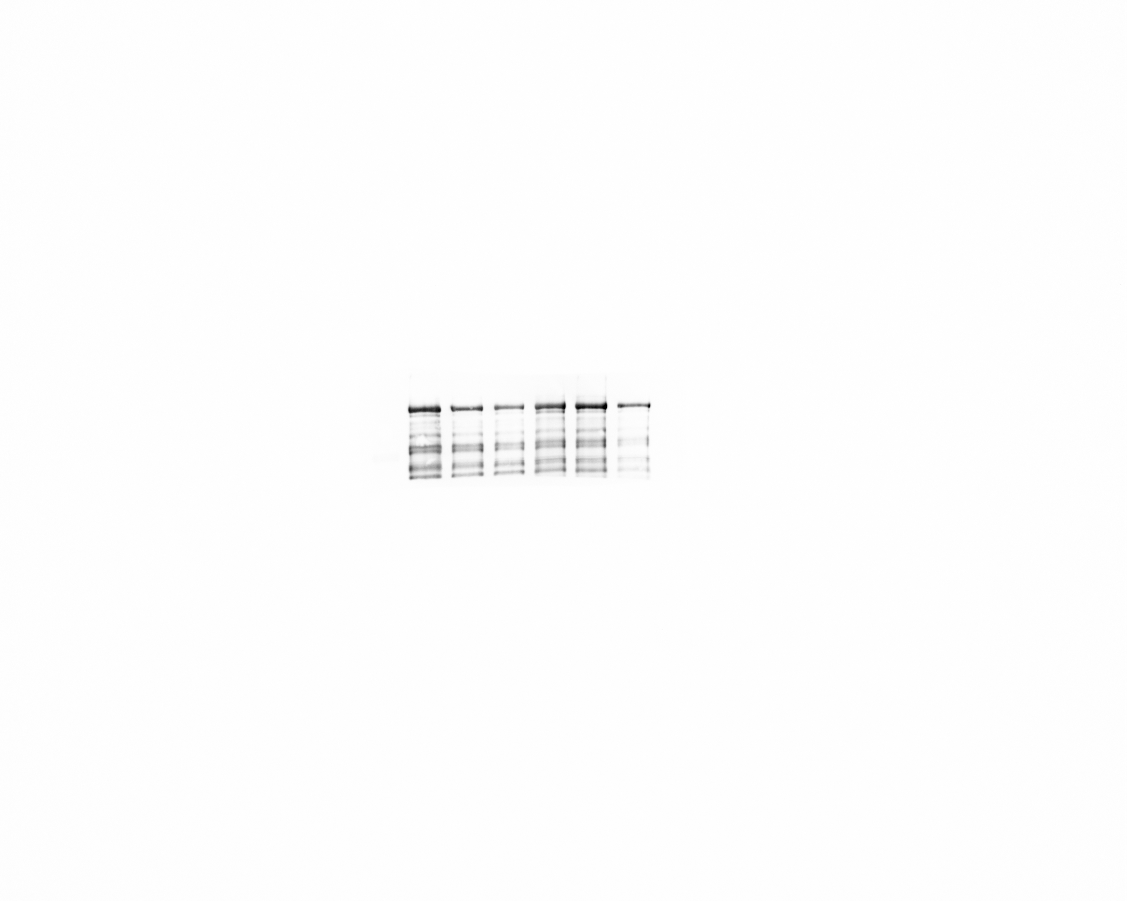

Supplement: Figure 4—source data 4. [file elife-81717-fig4-data4.zip › Figure 4 - Source Data 4 - 4G/Figure 4G- loading.tif]

**Figure 4H**

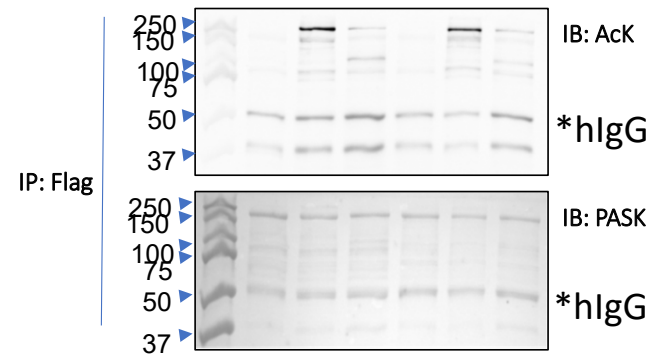

Supplement: Figure 4—source data 5. [file elife-81717-fig4-data5.zip › Figure 4 - Source Data 5- 4H/Figure 4H - Source Data.pdf]

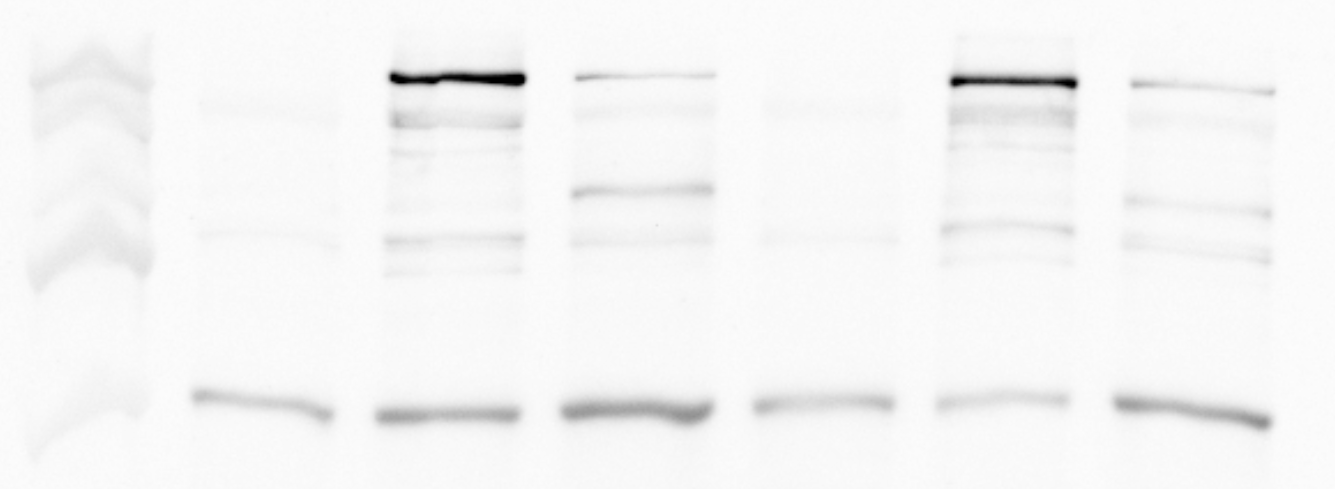

Supplement: Figure 4—source data 5. [file elife-81717-fig4-data5.zip › Figure 4 - Source Data 5- 4H/IB_AcK.tif]

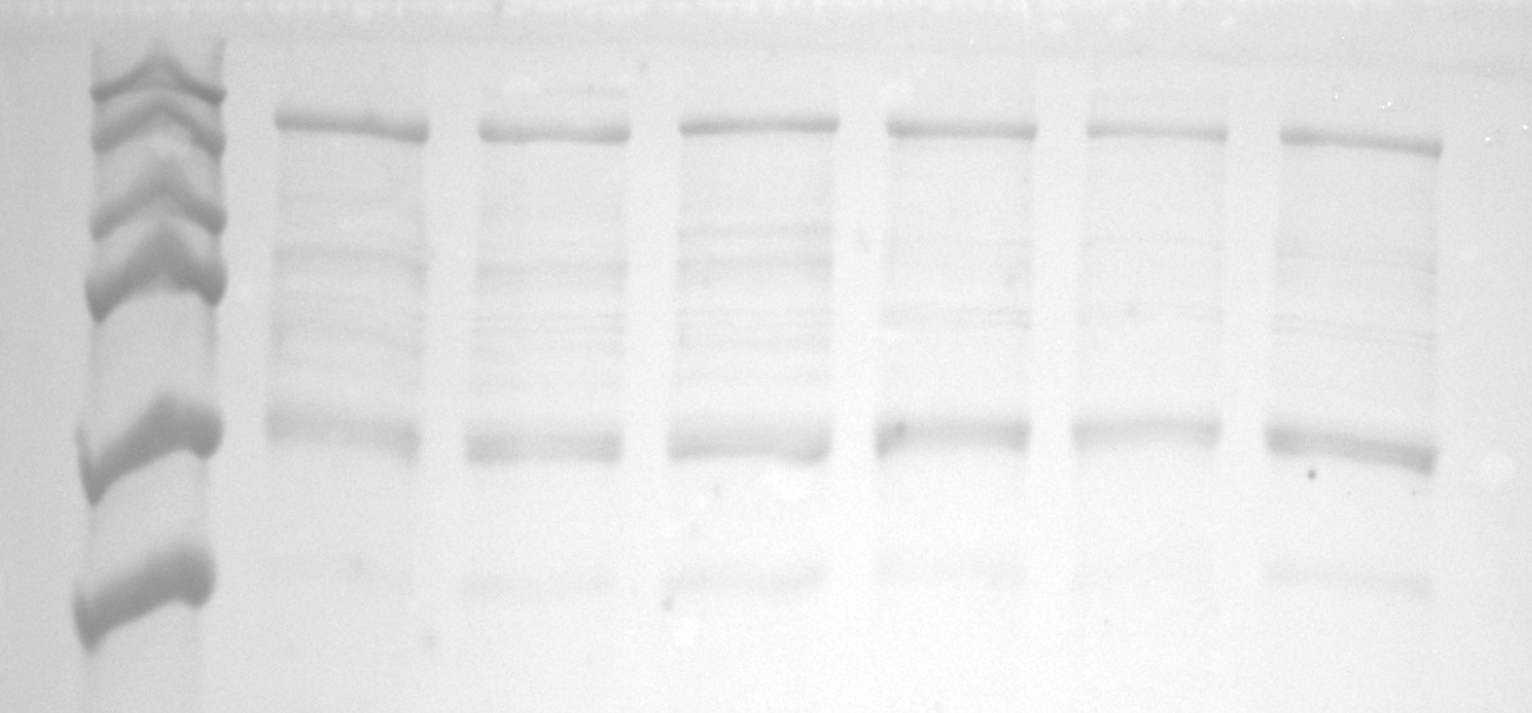

Supplement: Figure 4—source data 5. [file elife-81717-fig4-data5.zip › Figure 4 - Source Data 5- 4H/IB_PASK.tif]

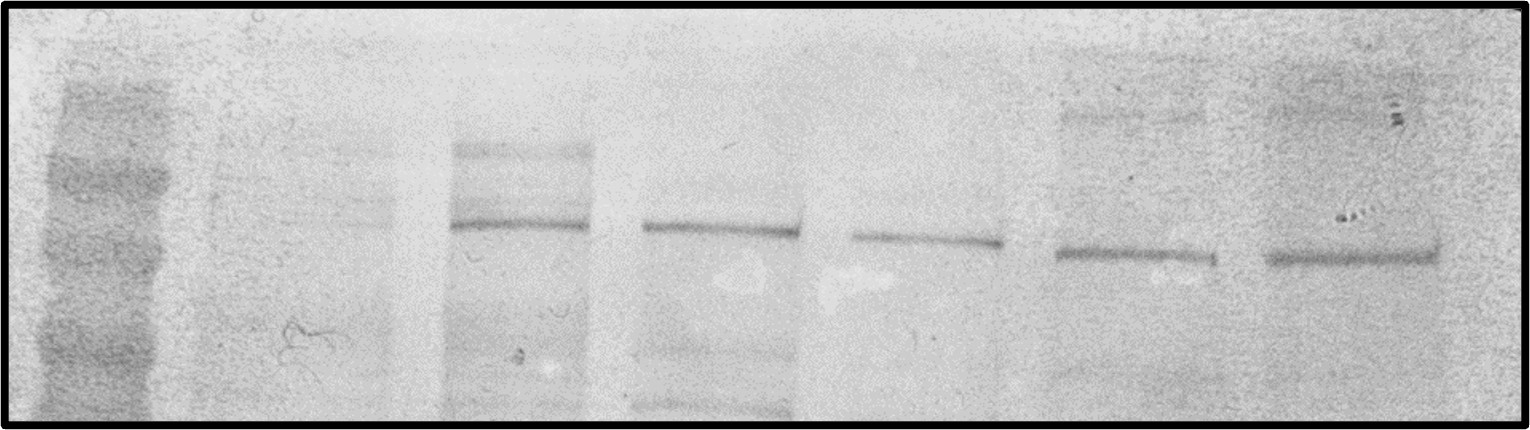

Supplement: Figure 4—source data 6. [file elife-81717-fig4-data6.zip › Figure 4 - Source Data 6 - S4B/anti PASK.jpg]

**Figure 4 – figure supplement 1B**

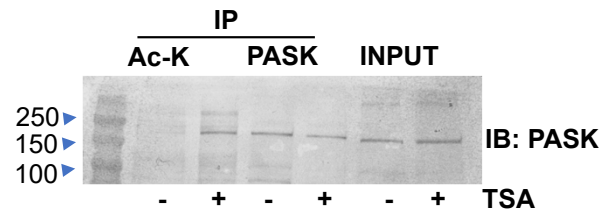

Supplement: Figure 4—source data 6. [file elife-81717-fig4-data6.zip › Figure 4 - Source Data 6 - S4B/Figure 4 - figure supplement 1B - Source Data.pdf]

**Figure 4 – figure supplement 1D**

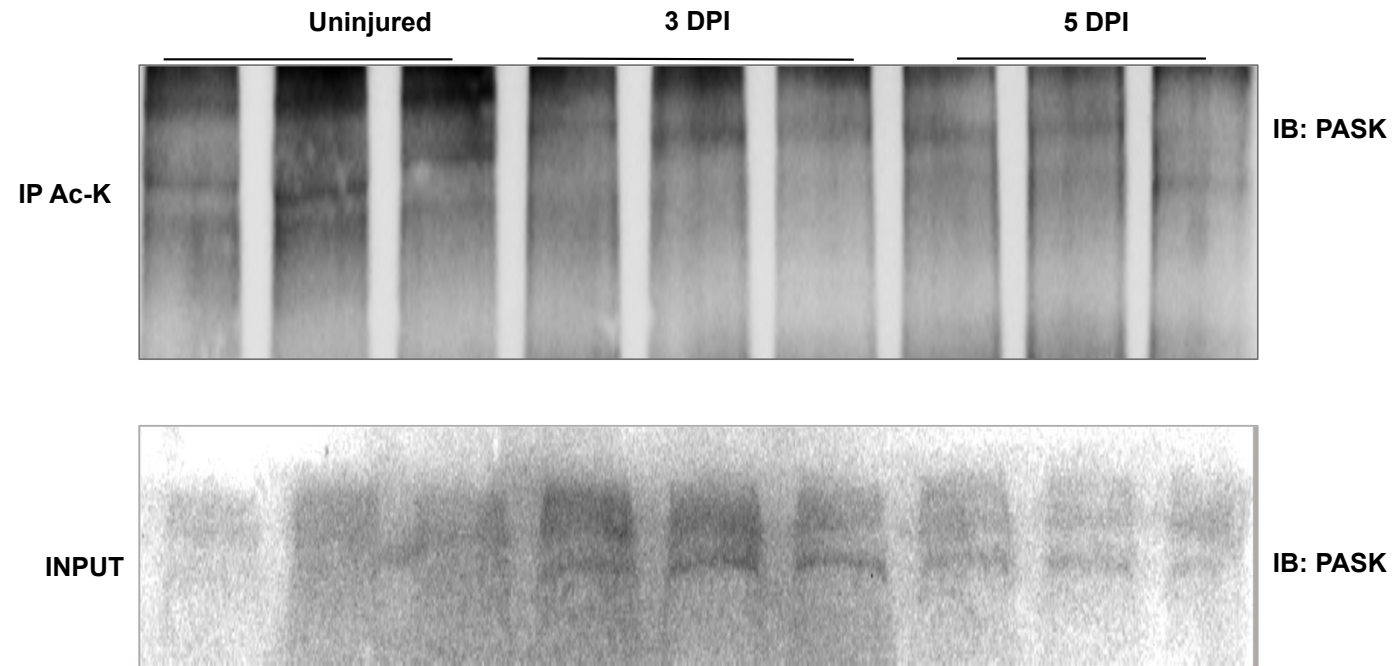

Supplement: Figure 4—figure supplement 1—source data 1. [file elife-81717-fig4-figsupp1-data1.zip › Figure 4 - Source Data 7 - S4D/Figure 4 - figure supplement 1D - Source Data.pdf]

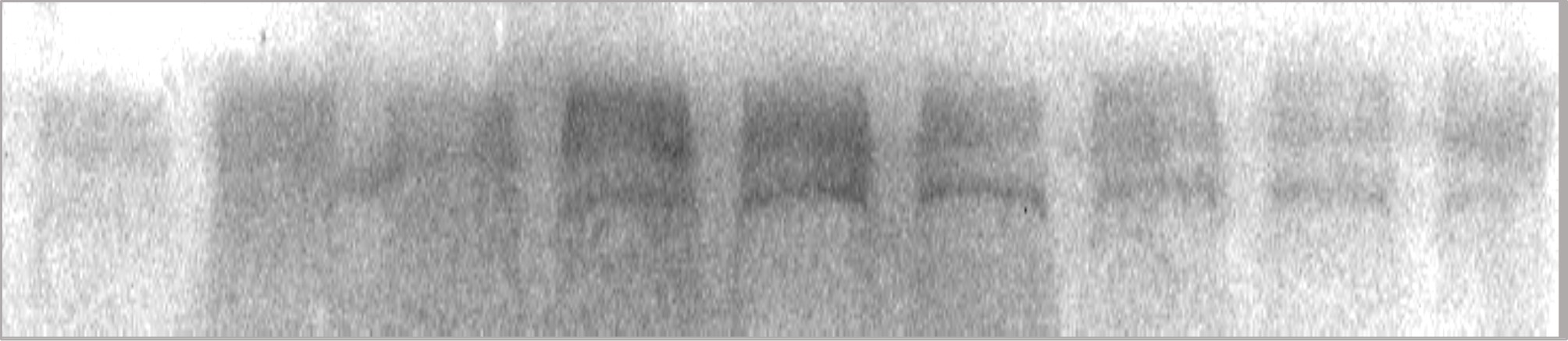

Supplement: Figure 4—figure supplement 1—source data 1. [file elife-81717-fig4-figsupp1-data1.zip › Figure 4 - Source Data 7 - S4D/InputPSK.jpg]

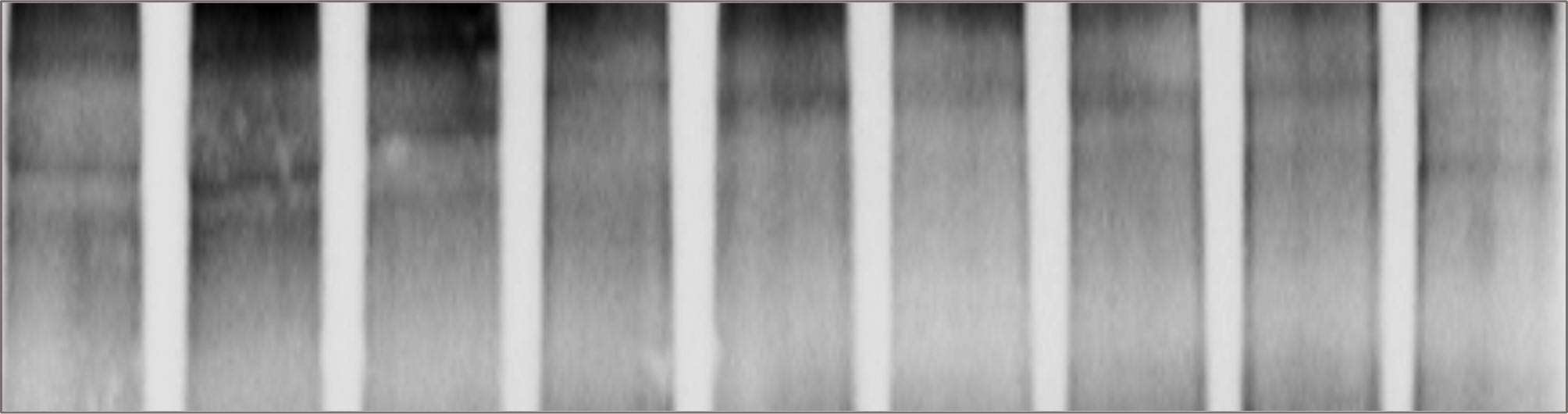

Supplement: Figure 4—figure supplement 1—source data 1. [file elife-81717-fig4-figsupp1-data1.zip › Figure 4 - Source Data 7 - S4D/IPAckWBPsk.jpg]

**Figure 4 – figure supplement 1C**

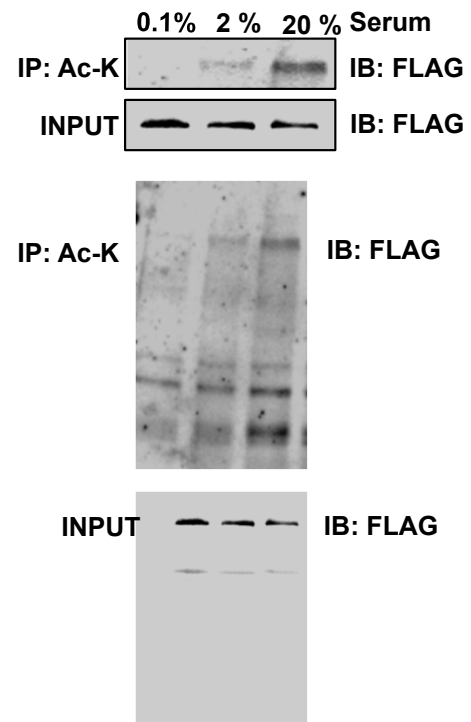

Supplement: Figure 4—figure supplement 1—source data 2. [file elife-81717-fig4-figsupp1-data2.zip › Figure 4 - Source Data 8 - S4C/Figure 4 - figure supplement 1C - Source Data.pdf]

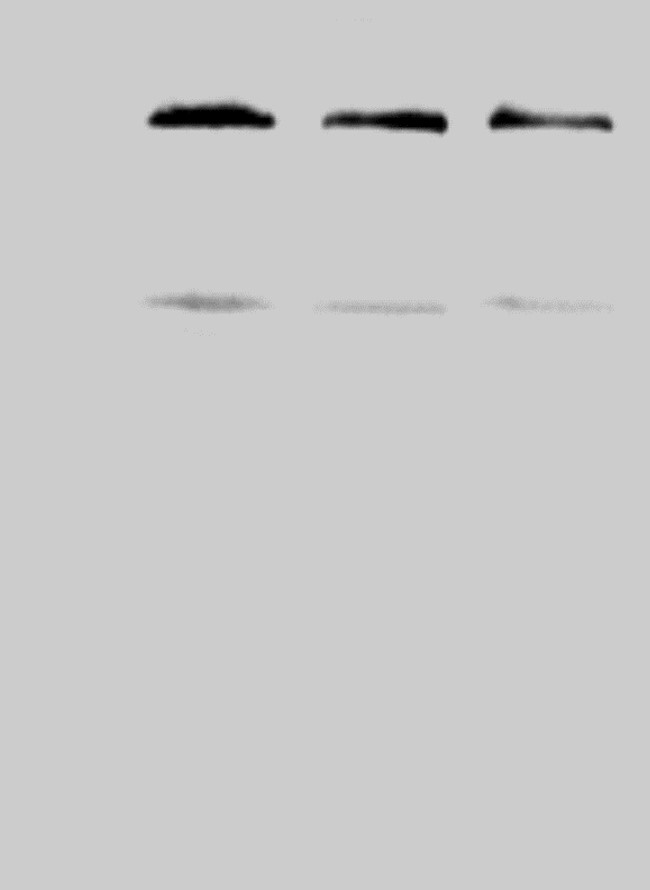

Supplement: Figure 4—figure supplement 1—source data 2. [file elife-81717-fig4-figsupp1-data2.zip › Figure 4 - Source Data 8 - S4C/IBFlg.jpg]

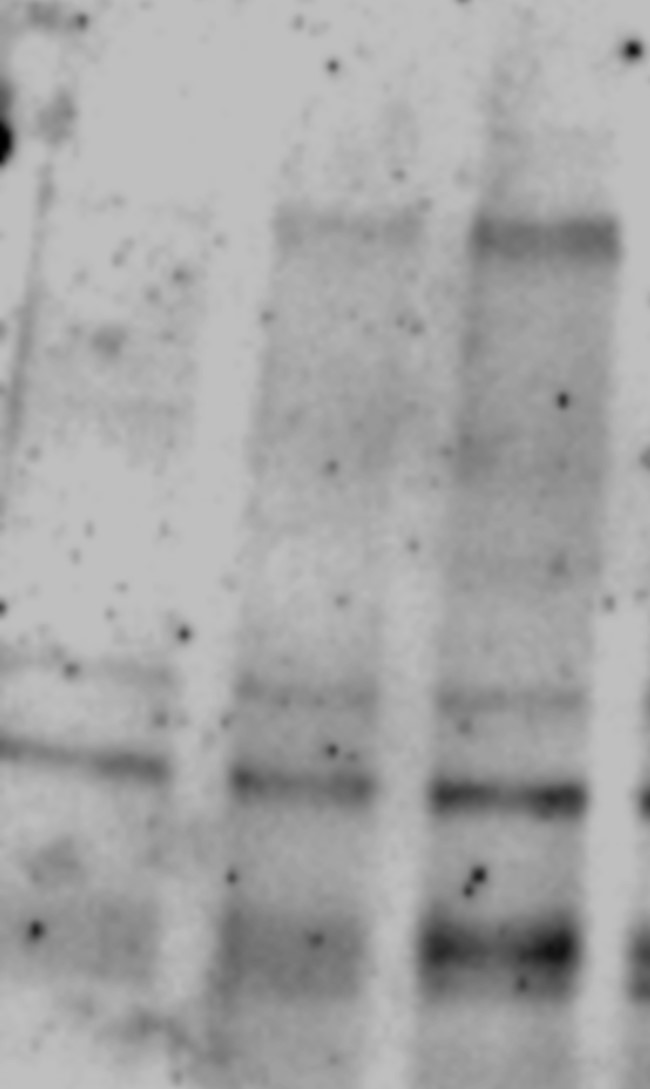

Supplement: Figure 4—figure supplement 1—source data 2. [file elife-81717-fig4-figsupp1-data2.zip › Figure 4 - Source Data 8 - S4C/IpAckWBFlg.jpg]

Figure 4 – figure supplement 1E

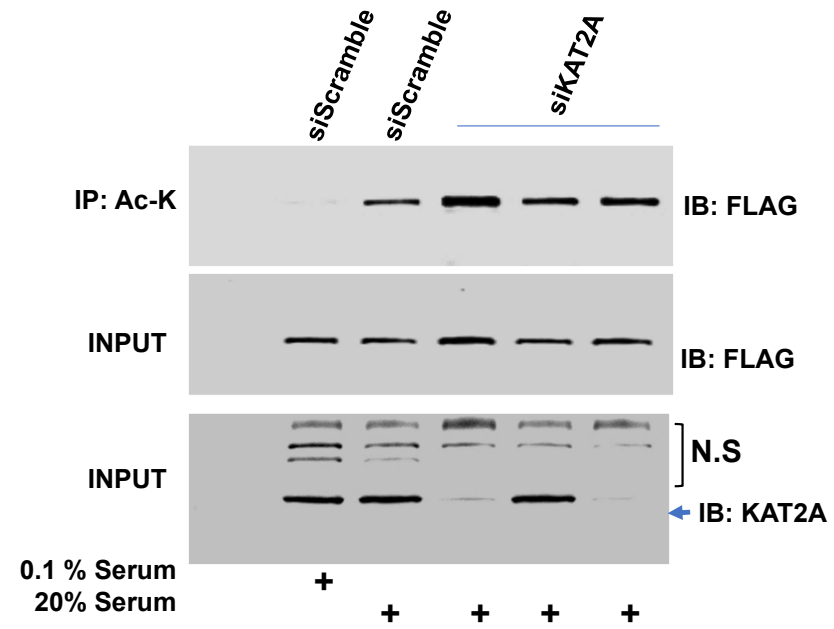

Supplement: Figure 4—figure supplement 1—source data 3. [file elife-81717-fig4-figsupp1-data3.zip › Figure 4 - Source Data 9 - S4E/Figure 4 - figure supplement 1E - Source Data.pdf]

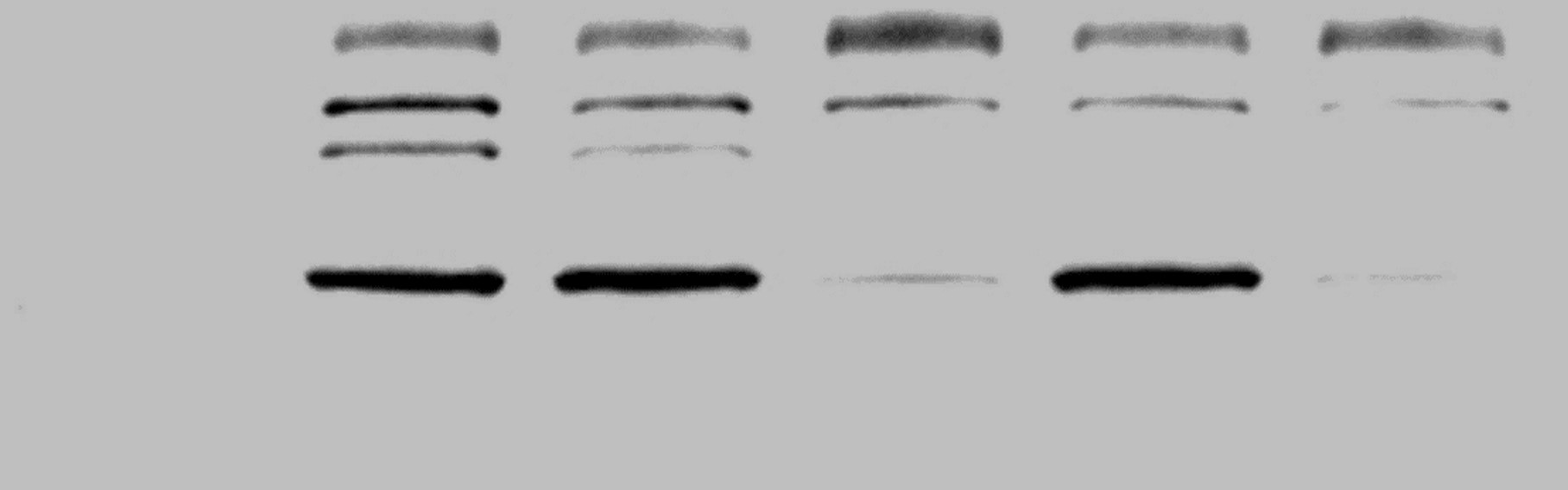

Supplement: Figure 4—figure supplement 1—source data 3. [file elife-81717-fig4-figsupp1-data3.zip › Figure 4 - Source Data 9 - S4E/Input_HATsOFF.jpg]

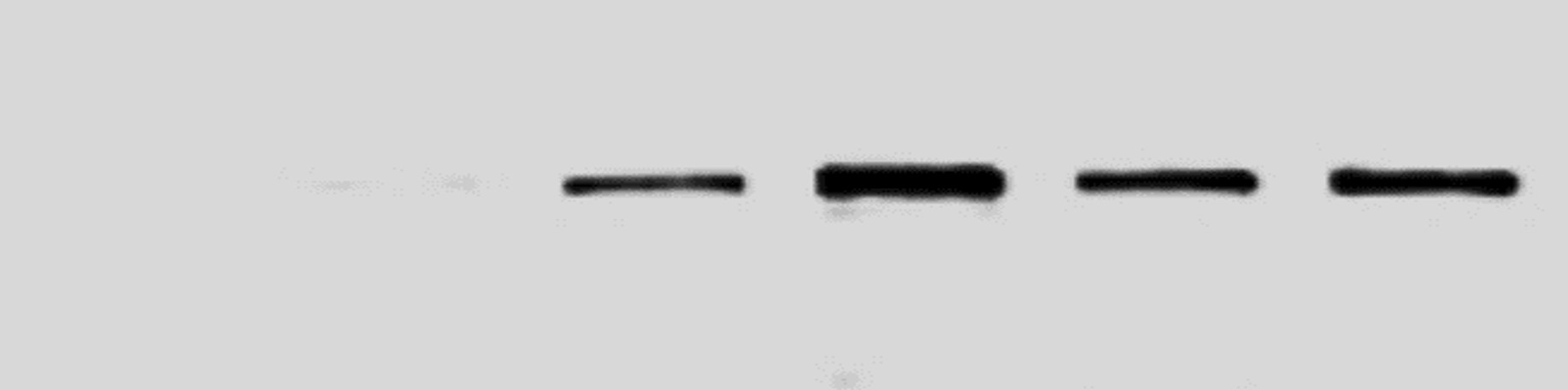

Supplement: Figure 4—figure supplement 1—source data 3. [file elife-81717-fig4-figsupp1-data3.zip › Figure 4 - Source Data 9 - S4E/IPAckWBFlg_1.jpg]

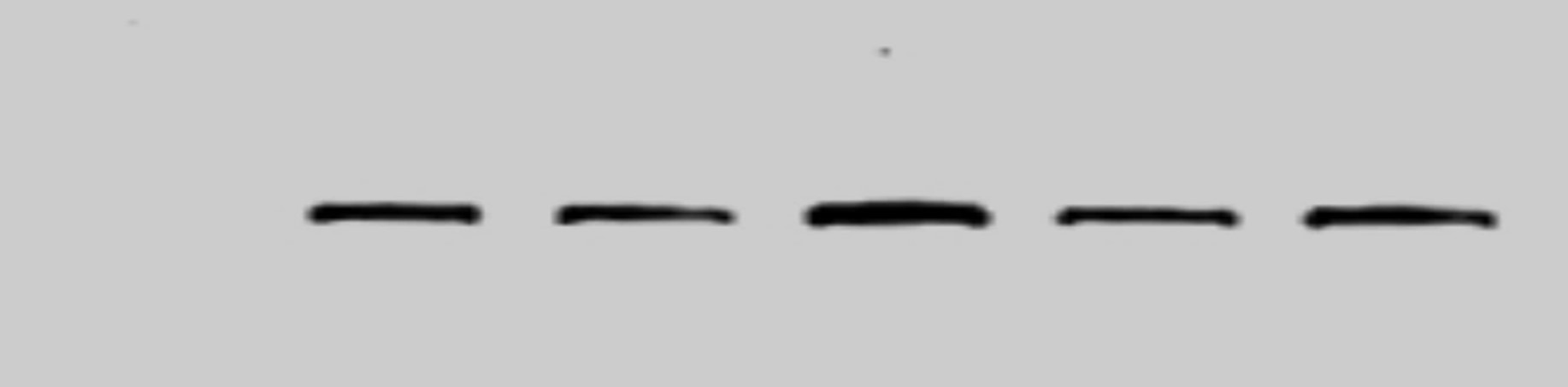

Supplement: Figure 4—figure supplement 1—source data 3. [file elife-81717-fig4-figsupp1-data3.zip › Figure 4 - Source Data 9 - S4E/TotalFlg_1.jpg]

Figure 4 – figure supplement 1F

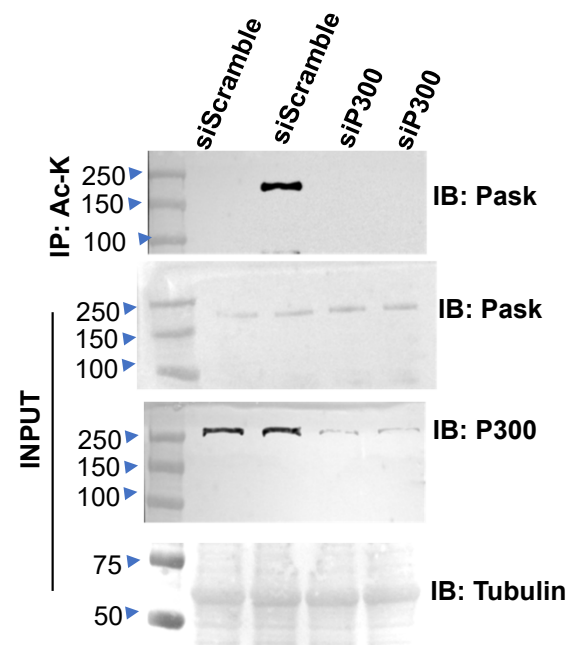

Supplement: Figure 4—figure supplement 1—source data 4. [file elife-81717-fig4-figsupp1-data4.zip › Figure 4 - Source Data 10 - S4F/Figure 4 - figure supplement 1F - Source Data.pdf]

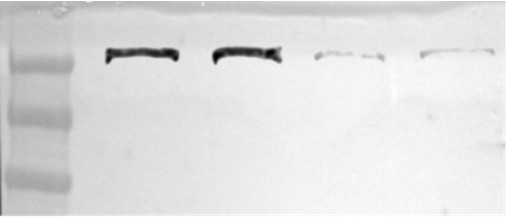

Supplement: Figure 4—figure supplement 1—source data 4. [file elife-81717-fig4-figsupp1-data4.zip › Figure 4 - Source Data 10 - S4F/Inputp300.jpg]

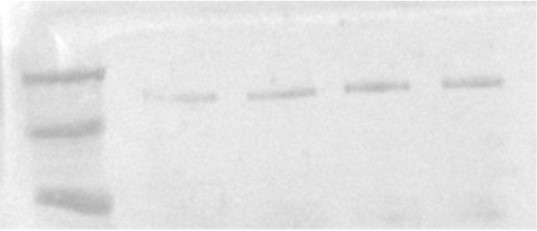

Supplement: Figure 4—figure supplement 1—source data 4. [file elife-81717-fig4-figsupp1-data4.zip › Figure 4 - Source Data 10 - S4F/InputPSK.jpg]

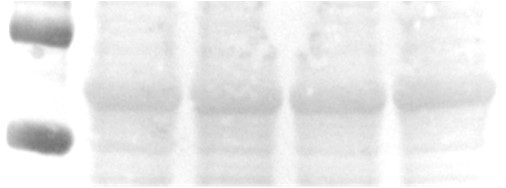

Supplement: Figure 4—figure supplement 1—source data 4. [file elife-81717-fig4-figsupp1-data4.zip › Figure 4 - Source Data 10 - S4F/inputTubload.jpg]

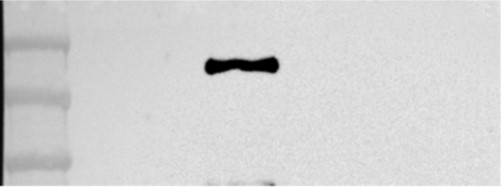

Supplement: Figure 4—figure supplement 1—source data 4. [file elife-81717-fig4-figsupp1-data4.zip › Figure 4 - Source Data 10 - S4F/IPAcK-WbPSK.jpg]

**Figure 4 – figure supplement 1G**

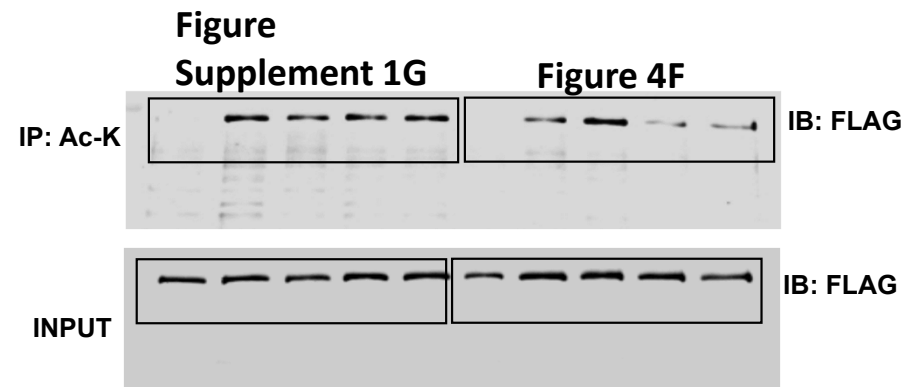

Supplement: Figure 4—figure supplement 1—source data 5. [file elife-81717-fig4-figsupp1-data5.zip › Figure 4 - Source Data 11 - S4G/Figure 4 - figure supplement 1G - Source Data.pdf]

**Figure 7B**

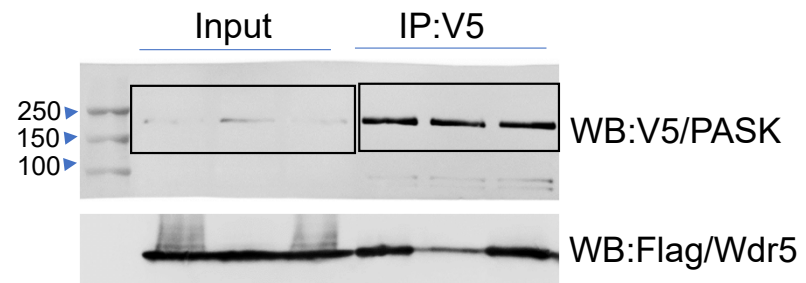

Supplement: Figure 7—source data 1. [file elife-81717-fig7-data1.zip › Figure 7 - Source Data 1/Figure 7B - Source Data.pdf]

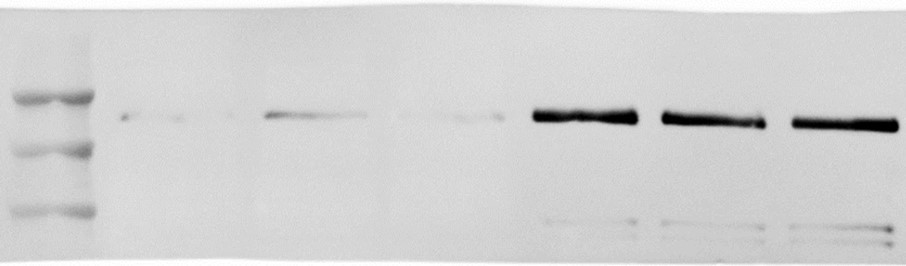

Supplement: Figure 7—source data 1. [file elife-81717-fig7-data1.zip › Figure 7 - Source Data 1/Pask.jpg]

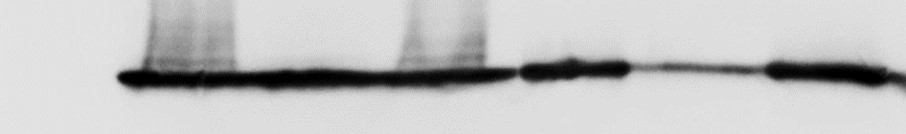

Supplement: Figure 7—source data 1. [file elife-81717-fig7-data1.zip › Figure 7 - Source Data 1/Wdr5_flg.jpg]

**Figure 7C**

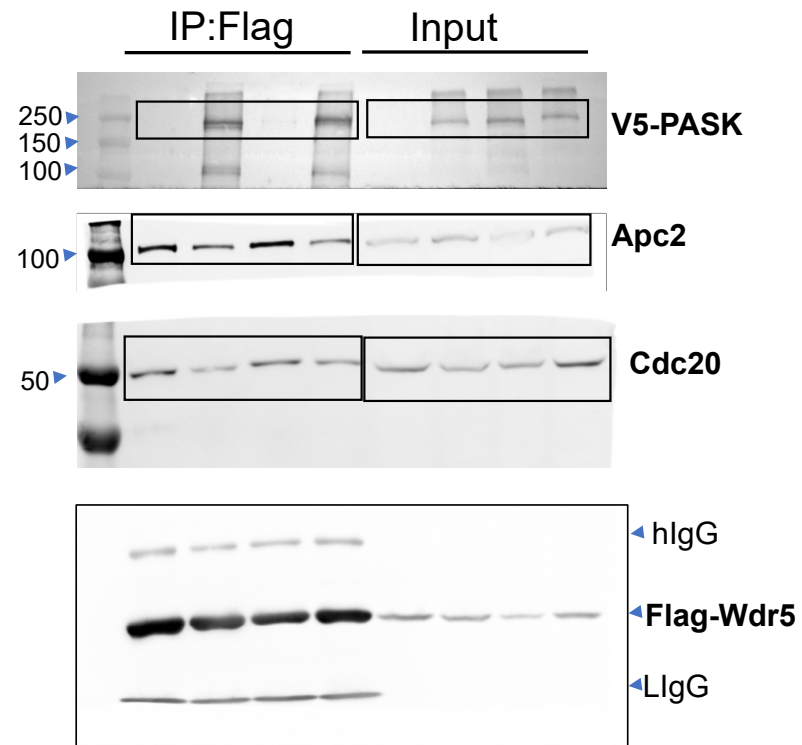

Supplement: Figure 7—source data 2. [file elife-81717-fig7-data2.zip › Figure 7 - Source Data 2/Figure 7C - Source Data.pdf]

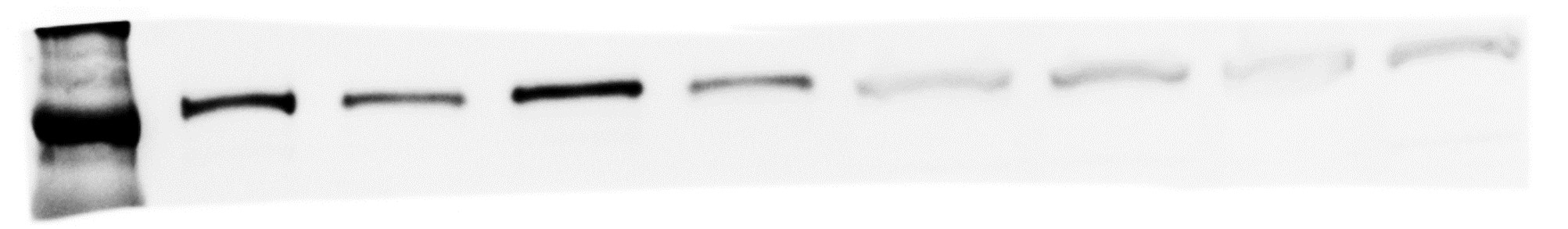

Supplement: Figure 7—source data 2. [file elife-81717-fig7-data2.zip › Figure 7 - Source Data 2/IPFlgWBApc2.jpg]

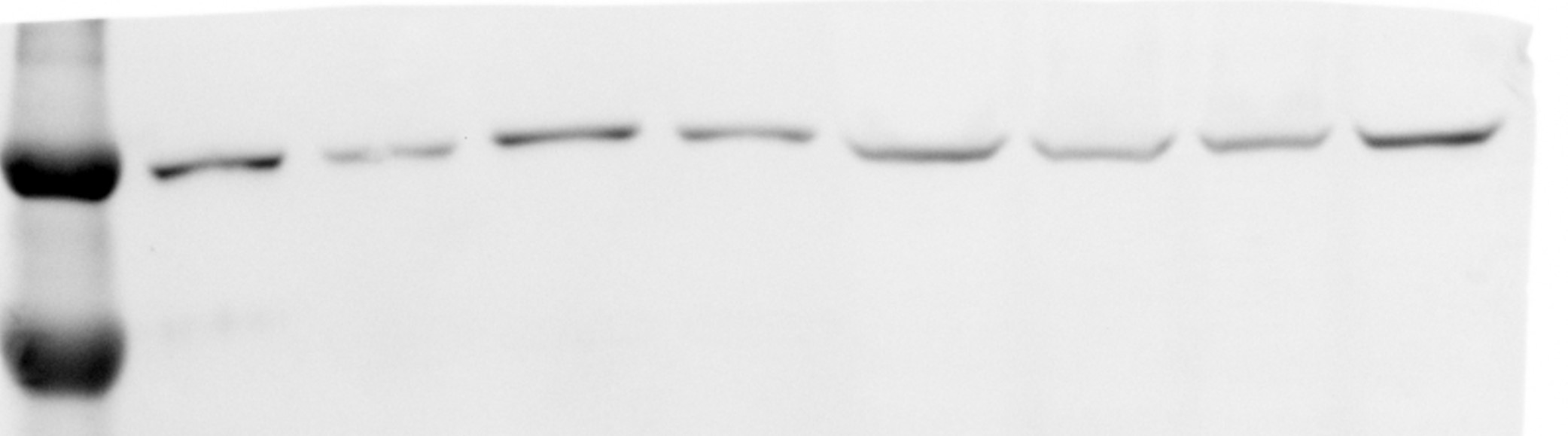

Supplement: Figure 7—source data 2. [file elife-81717-fig7-data2.zip › Figure 7 - Source Data 2/IPFlgWBcdc20.jpg]

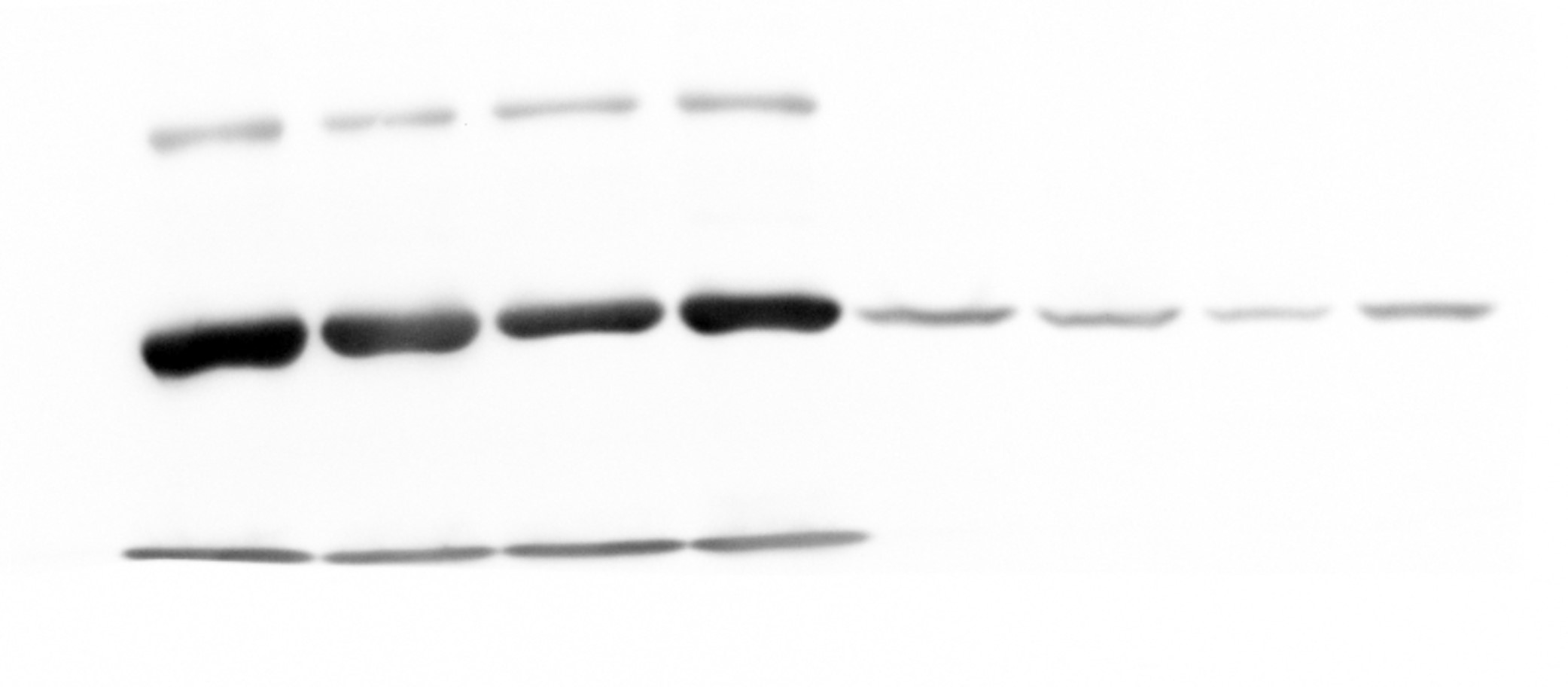

Supplement: Figure 7—source data 2. [file elife-81717-fig7-data2.zip › Figure 7 - Source Data 2/IPFlgWBFlg.jpg]

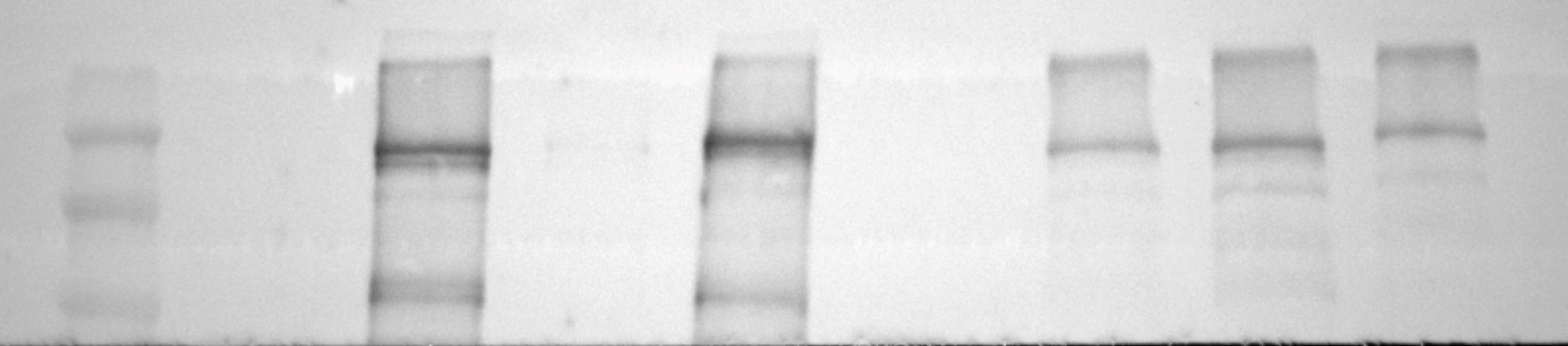

Supplement: Figure 7—source data 2. [file elife-81717-fig7-data2.zip › Figure 7 - Source Data 2/IPFlgWBV5.jpg]

**Figure 7D**

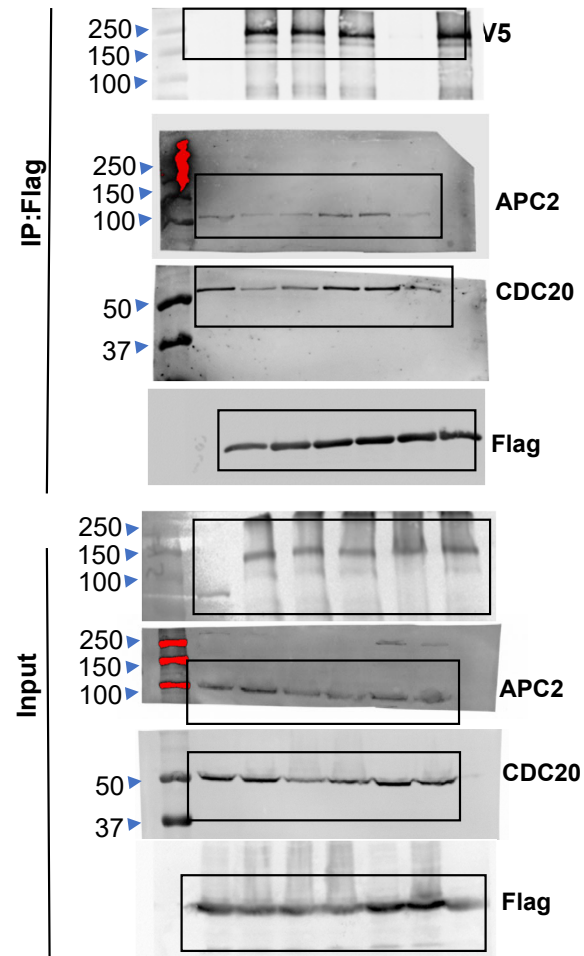

Supplement: Figure 7—source data 3. [file elife-81717-fig7-data3.zip › Figure 7 - Source Data 3/Figure 7D - Source Data.pdf]

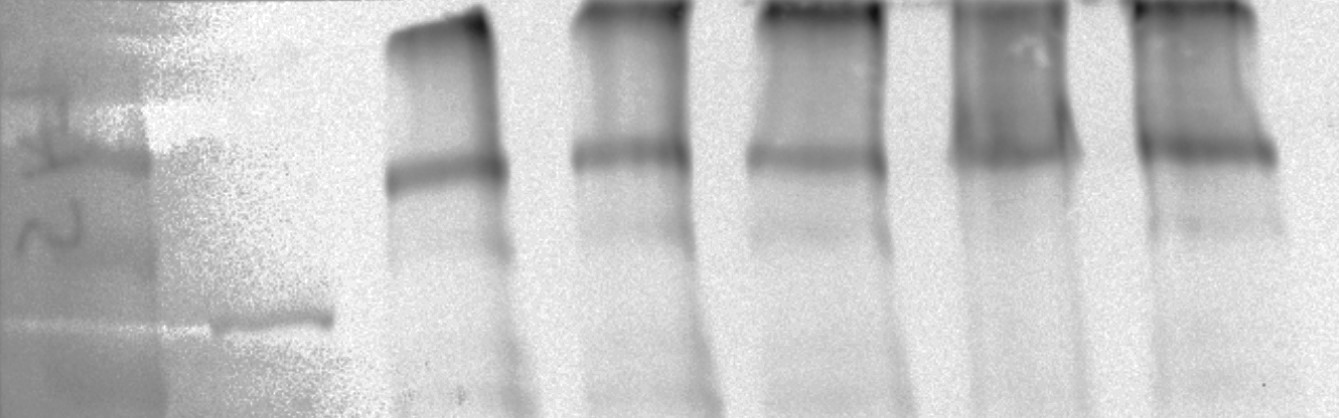

Supplement: Figure 7—source data 3. [file elife-81717-fig7-data3.zip › Figure 7 - Source Data 3/InputV5.jpg]

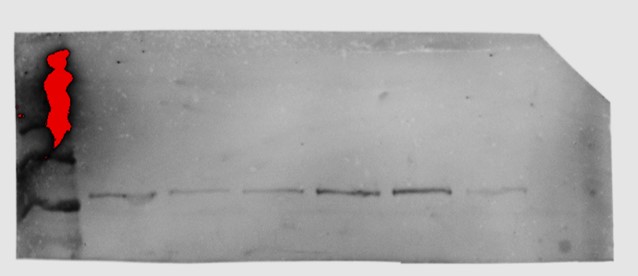

Supplement: Figure 7—source data 3. [file elife-81717-fig7-data3.zip › Figure 7 - Source Data 3/IPFlgWBApc2.jpg]

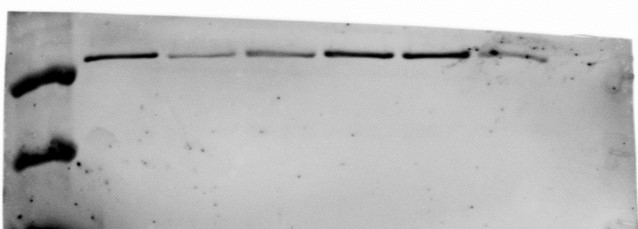

Supplement: Figure 7—source data 3. [file elife-81717-fig7-data3.zip › Figure 7 - Source Data 3/IPFlgWBcdc20.jpg]

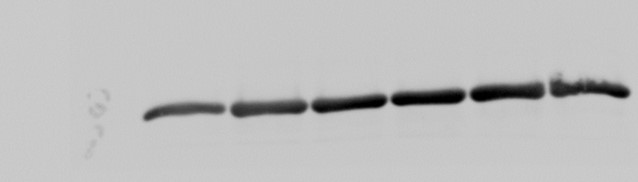

Supplement: Figure 7—source data 3. [file elife-81717-fig7-data3.zip › Figure 7 - Source Data 3/IPFlgWBFlg.jpg]

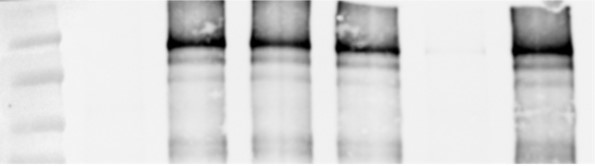

Supplement: Figure 7—source data 3. [file elife-81717-fig7-data3.zip › Figure 7 - Source Data 3/IPFlgWBV5.jpg]

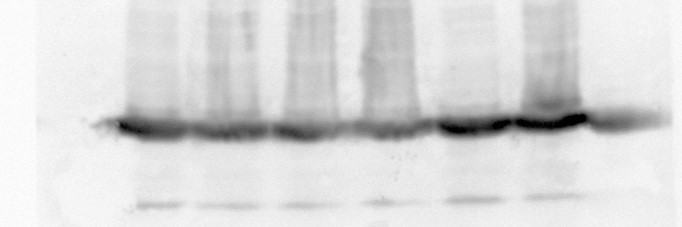

Supplement: Figure 7—source data 3. [file elife-81717-fig7-data3.zip › Figure 7 - Source Data 3/Total Flg.jpg]

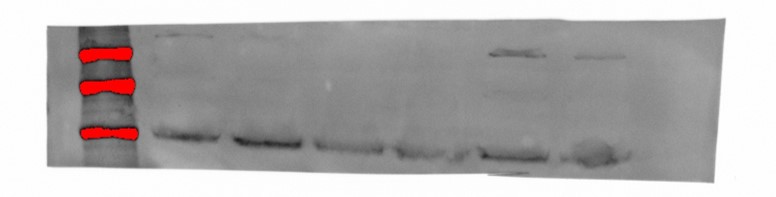

Supplement: Figure 7—source data 3. [file elife-81717-fig7-data3.zip › Figure 7 - Source Data 3/TotalApc2.jpg]

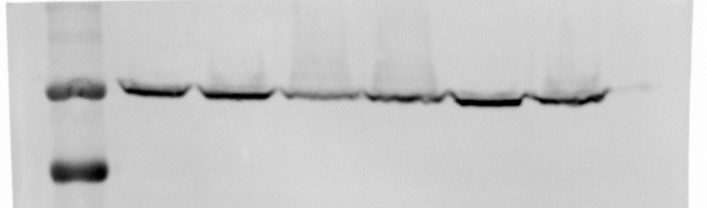

Supplement: Figure 7—source data 3. [file elife-81717-fig7-data3.zip › Figure 7 - Source Data 3/Totalcdc20.jpg]

**Figure 7E**

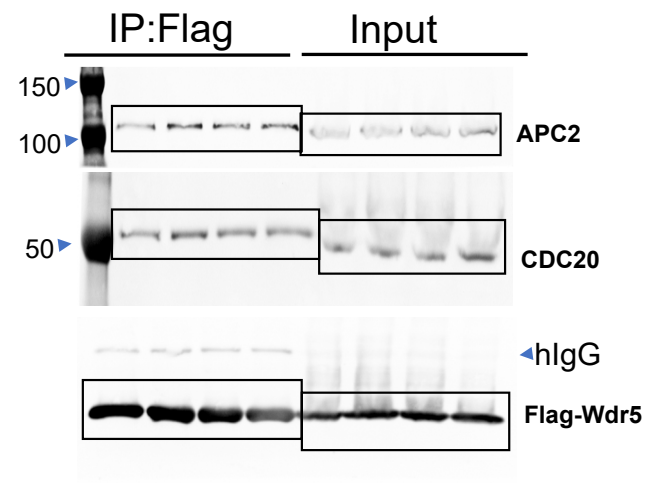

Supplement: Figure 7—source data 4. [file elife-81717-fig7-data4.zip › Figure 7 - Source Data 4/Figure 7E - Source Data.pdf]

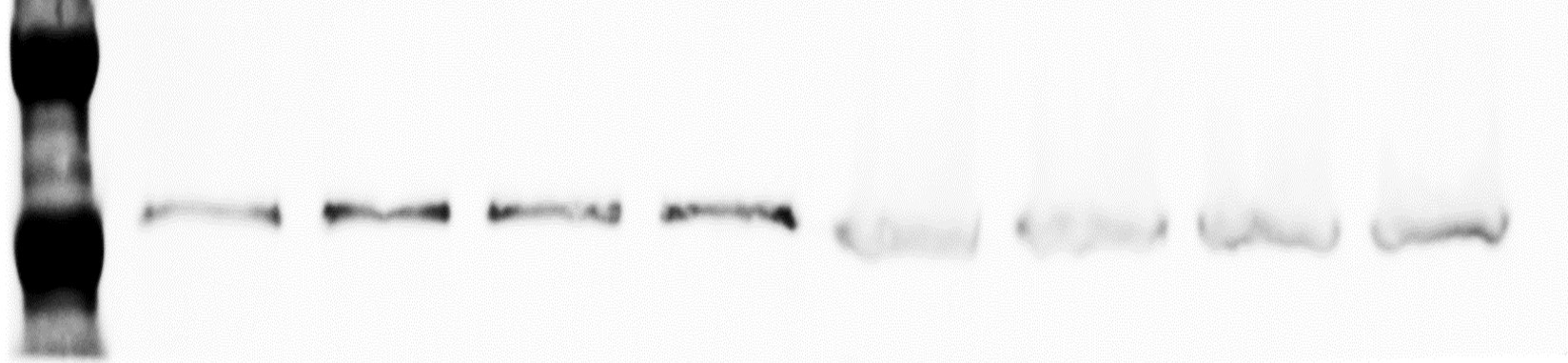

Supplement: Figure 7—source data 4. [file elife-81717-fig7-data4.zip › Figure 7 - Source Data 4/IPFlgWBApc2.jpg]

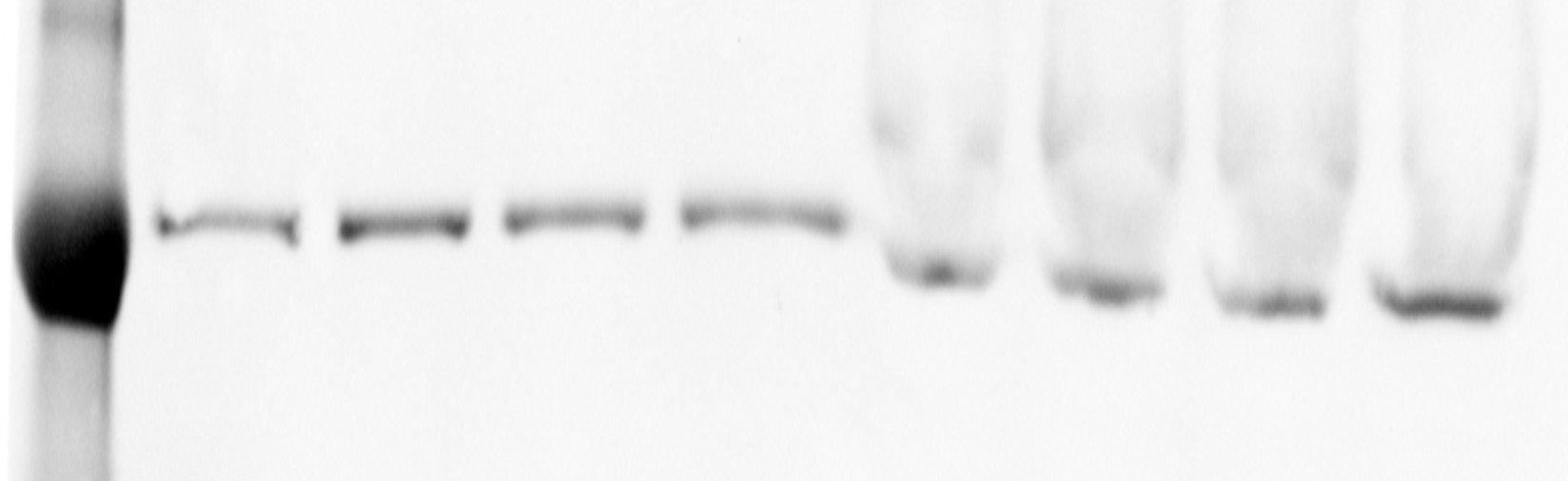

Supplement: Figure 7—source data 4. [file elife-81717-fig7-data4.zip › Figure 7 - Source Data 4/IPFlgWBcdc20.jpg]

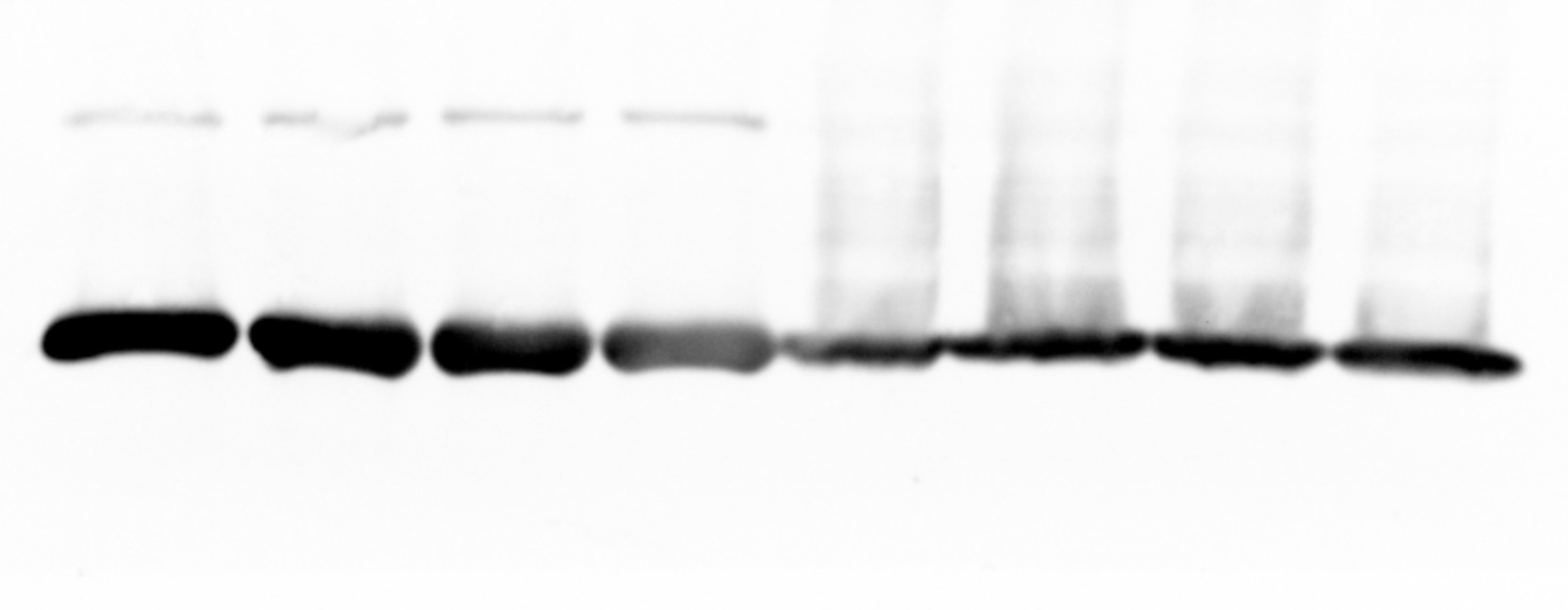

Supplement: Figure 7—source data 4. [file elife-81717-fig7-data4.zip › Figure 7 - Source Data 4/IPFlgWBFlg.jpg]

Figure 7 – figure supplement 1B

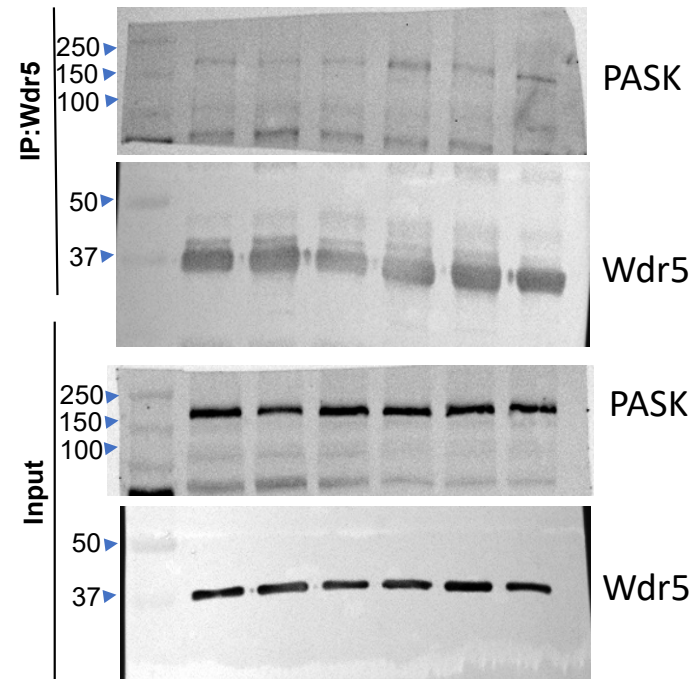

Supplement: Figure 7—figure supplement 1—source data 1. [file elife-81717-fig7-figsupp1-data1.zip › Figure 7 - figure supplement 1 - Source Data 1/Figure 7 - figure supplement 1B - Source Data.pdf]

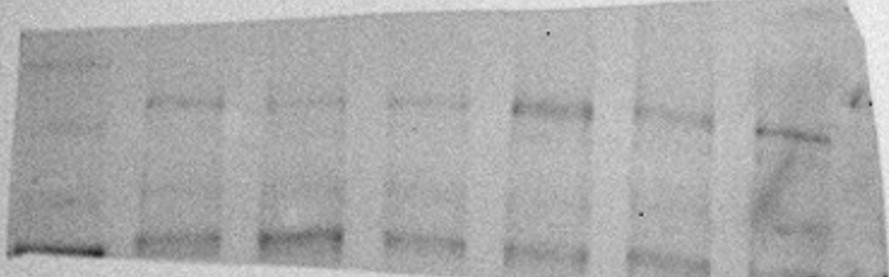

Supplement: Figure 7—figure supplement 1—source data 1. [file elife-81717-fig7-figsupp1-data1.zip › Figure 7 - figure supplement 1 - Source Data 1/IPWdr5WBpask.jpg]

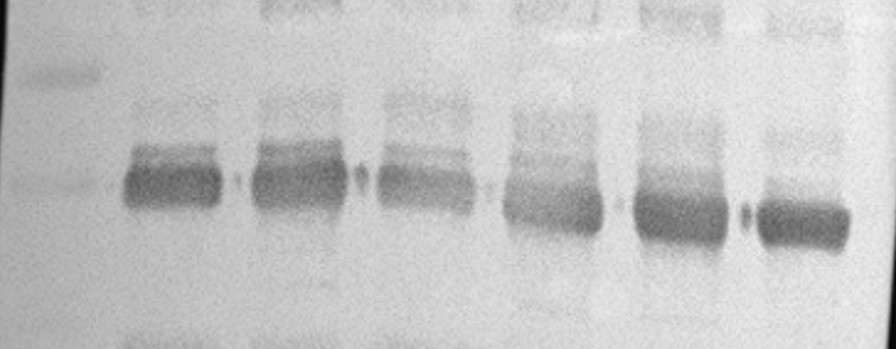

Supplement: Figure 7—figure supplement 1—source data 1. [file elife-81717-fig7-figsupp1-data1.zip › Figure 7 - figure supplement 1 - Source Data 1/IPWdr5WBWdr5.jpg]

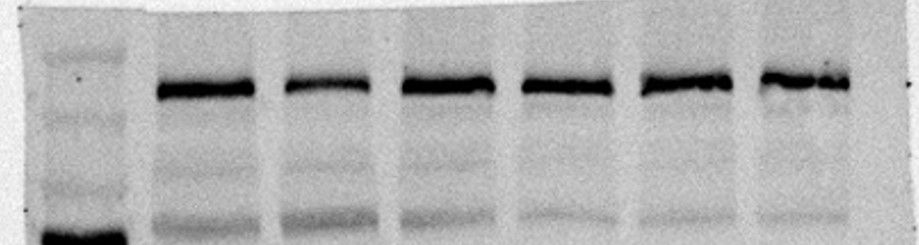

Supplement: Figure 7—figure supplement 1—source data 1. [file elife-81717-fig7-figsupp1-data1.zip › Figure 7 - figure supplement 1 - Source Data 1/TotalPASK.jpg]

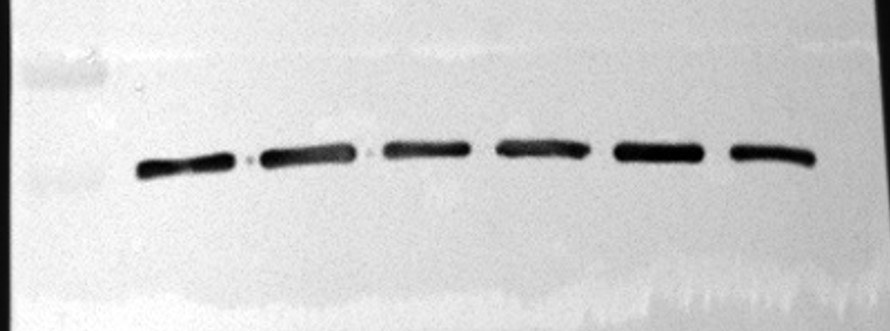

Supplement: Figure 7—figure supplement 1—source data 1. [file elife-81717-fig7-figsupp1-data1.zip › Figure 7 - figure supplement 1 - Source Data 1/TotalWdr5.jpg]

Figure 7 – figure supplement 1C

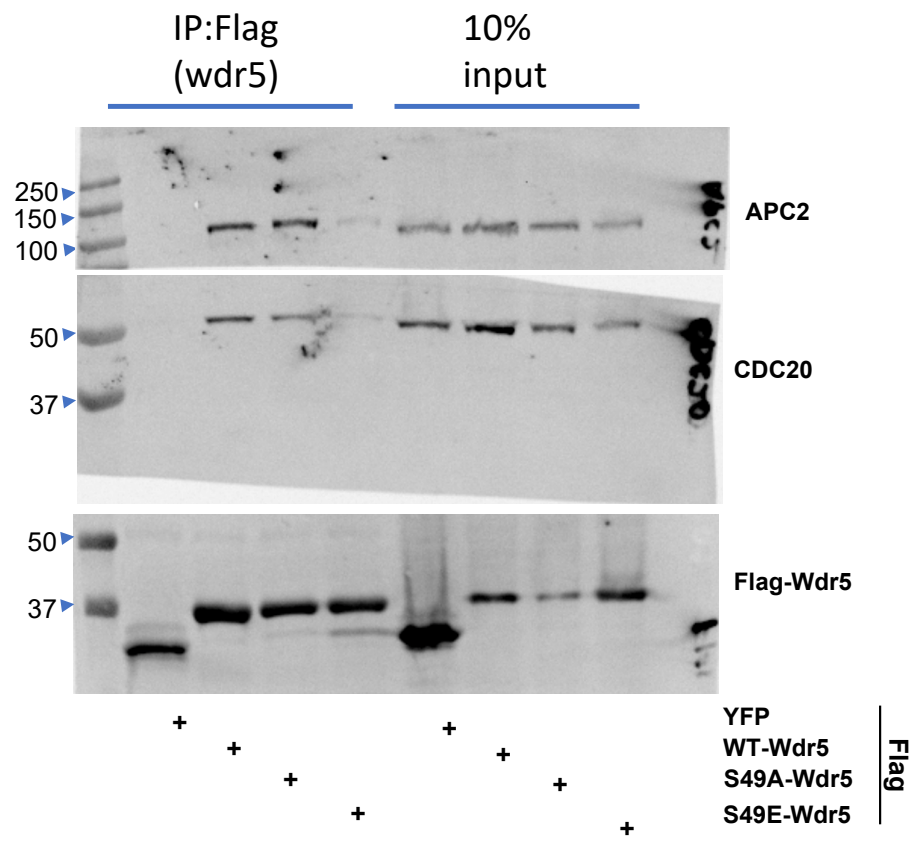

Supplement: Figure 7—figure supplement 1—source data 2. [file elife-81717-fig7-figsupp1-data2.zip › Figure 7 - figure supplement 1-Source Data - 2/Figure 7 - figure supplement 1C - Source Data.pdf]

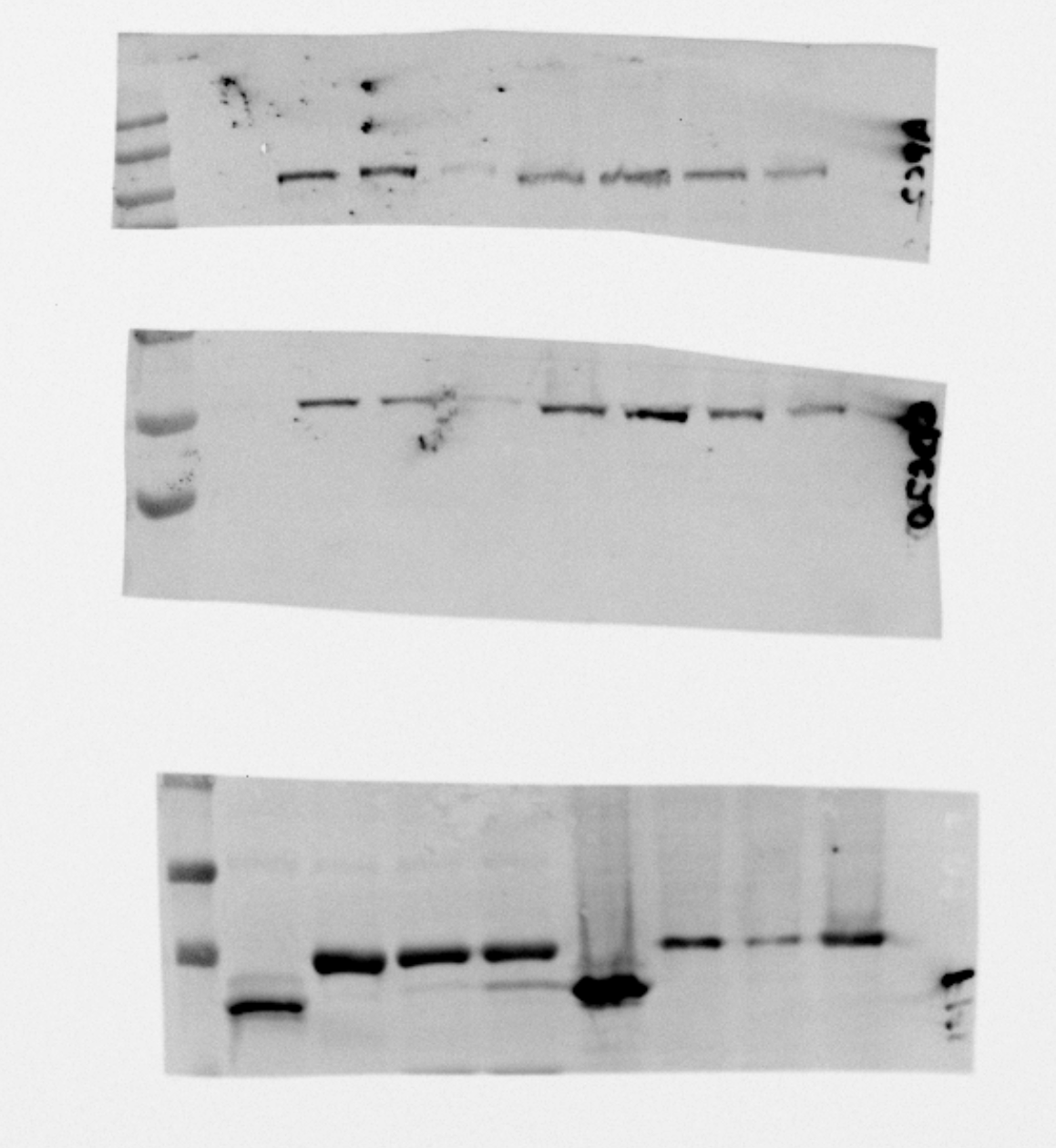

Supplement: Figure 7—figure supplement 1—source data 2. [file elife-81717-fig7-figsupp1-data2.zip › Figure 7 - figure supplement 1-Source Data - 2/Raw WB Data.tif]
