# Supplementary material for: PASK links cellular energy metabolism with a mitotic self-renewal network to establish differentiation competence
Source: eLife. 2023 Apr 13;12:e81717. doi: 10.7554/eLife.81717 (PMC10162801; doi:10.7554/eLife.81717)
Supplement: Figure 3—source data 3. [file elife-81717-fig3-data3.zip › Figure 3 - Source Data 3/Figure 3 - Figure supplement 3.docx]

**Figure 3 – Source Data Table 3**
