# Supplementary material for: PASK links cellular energy metabolism with a mitotic self-renewal network to establish differentiation competence
Source: eLife. 2023 Apr 13;12:e81717. doi: 10.7554/eLife.81717 (PMC10162801; doi:10.7554/eLife.81717)

Figure 4G

Steady-state levels of ac-and total – PASK. Not included in the final figure but is a useful control for ratio of ac/total PASK between lanes 1 and 4-5.

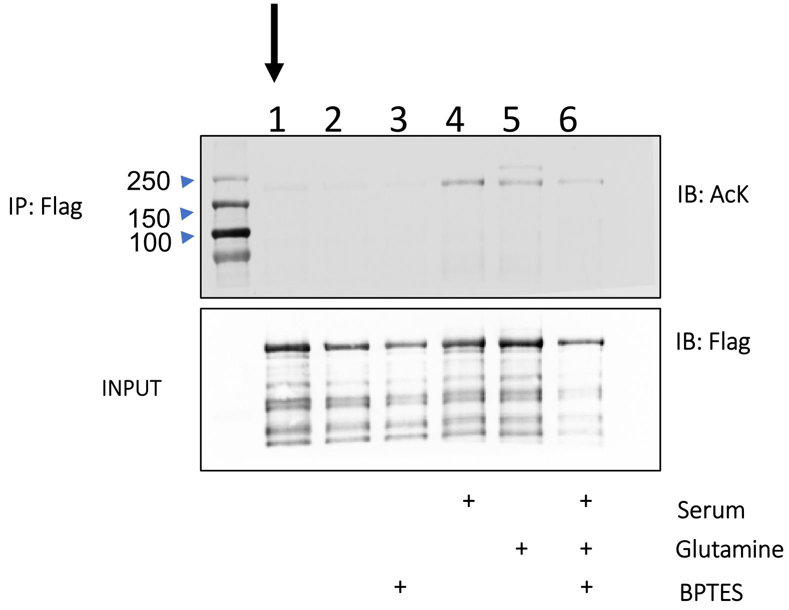

Supplement: Figure 4—source data 4. [file elife-81717-fig4-data4.zip › Figure 4 - Source Data 4 - 4G/Figure 4G - Source Data.pdf]
