# Supplementary material for: PASK links cellular energy metabolism with a mitotic self-renewal network to establish differentiation competence
Source: eLife. 2023 Apr 13;12:e81717. doi: 10.7554/eLife.81717 (PMC10162801; doi:10.7554/eLife.81717)
Supplement: Supplementary file 1. [file elife-81717-supp1.docx]

Supplementary File 1: Plasmids available upon request

| **Plasmid ID** | **Description** | **Mammalian cell drug selection** | **Bacterial cell drug selection** |
| --- | --- | --- | --- |
| pCDNA.A/hPASK-V5 | A non-viral mammalian expression vector to express V5-Tag human PASK |  | Ampicillin |
| pCDNA.A/hPASK-V5-NES1 | A non-viral mammalian expression vector to express V5-Tag human PASK with NES1 mutation (see text for information about the residues) |  | Ampicillin |
| pCDNA.A/hPASK-V5-NES2 | A non-viral mammalian expression vector to express V5-Tag human PASK with NES2 mutation (see text for information about the residues) |  | Ampicillin |
| pCDNA.A/hPASK-V5-NES1+NES2 | A non-viral mammalian expression vector to express V5-Tag human PASK with NES1 and NES2 mutation (see text for information about the residues) |  | Ampicillin |
| pCDNA.A/hPASK-V5-R942G | A non-viral mammalian expression vector to express V5-Tag human PASK with mutations in WIN motif (R942G) |  | Ampicillin |
| pQCXIP/hPASK-GFP | A retroviral mammalian expression vector to express V5-Tag human PASK | Puromycin | Ampicillin |
| pQCXIP/SV40-hPASK-V5-NES1+NES2-GFP | A retroviral mammalian expression vector to express V5-Tag human PASK with NES1 mutation (see text for information about the residues) | Puromycin | Ampicillin |
| pQCXIP/hPASK-V5-R942G-GFP | A retroviral mammalian expression vector to express V5-Tag human PASK with NES2 mutation (see text for information about the residues) | Puromycin | Ampicillin |
| pQCXIP/SV40-hPASK-V5-NES1+NES2-R942G-GFP | A retroviral mammalian expression vector to express V5-Tag human PASK with NES1 and NES2 mutation (see text for information about the residues) | Puromycin | Ampicillin |
